# Supplementary material for: Non-covalent ligand-oxide interaction promotes oxygen evolution
Source: Nat Commun. 2023 Feb 22;14:997. doi: 10.1038/s41467-023-36718-3 (PMC9947139; doi:10.1038/s41467-023-36718-3)
Supplement: Supplementary file 1 — Supplementary Information [file 41467_2023_36718_MOESM1_ESM.pdf]

Supplementary Information

Non-covalent ligand-oxide interaction promotes oxygen evolution

Qianbao Wu[1, ‡], Junwu Liang[2, ‡], Mengjun Xiao[1, ‡], Chang Long[1], Lei Li[1], Zhenhua Zeng[3, *], Andraž Mavrič[4], Xia Zheng[1], Jing Zhu[5], Hai-Wei Liang[6], Hongfei Liu[1], Matjaz Valant[4], Wei Wang[7], Zhengxing Lv[8], Jiong Li[8], Chunhua Cui[1,*]

[1]Molecular Electrochemistry Laboratory, Institute of Fundamental and Frontier Sciences, University of Electronic Science and Technology of China, Chengdu, 610054, China.

[2]Optoelectronic Information Research Center, School of Physics and Telecommunication Engineering, Yulin Normal University, Yulin, Guangxi, 537000, China.

[3]Davidson School of Chemical Engineering, Purdue University, West Lafayette, Indiana 47907, United States.

[4]Materials Research Laboratory, University of Nova Gorica, Vipavska 13, SI-5000 Nova Gorica, Slovenia.

[5]Department of Chemical Physics, School of Chemistry and Materials Science, University of Science and Technology of China, Hefei, 230026, China.

[6]Hefei National Laboratory for Physical Sciences at the Microscale, Department of Chemistry, University of Science and Technology of China, Hefei, 230026, China.

[7]School of Materials and Energy, University of Electronic Science and Technology of China, Chengdu, 610054, China.

[8]Shanghai Synchrotron Radiation Facility, Shanghai Advanced Research Institute, Chinese Academy of Sciences, Shanghai, 201210 P. R. China

[*]E-mail: zeng46@purdue.edu, chunhua.cui@uestc.edu.cn

[‡]These authors contribute equally

This file concludes:

Supplementary Fig. 1 to 69

Supplementary Table 1 to 10

Supplementary References 1 to 44

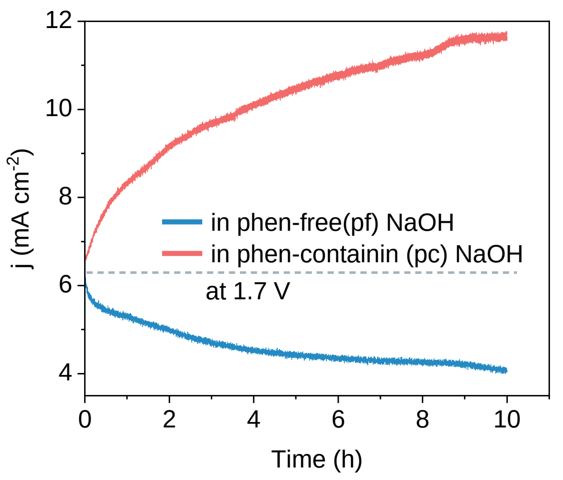

Supplementary Fig. 1 | *j*-t curves. The *j*-*t* curves of as-prepared CoOOH on FTO at 1.7 V_{RHE} in phen-free (pf-) and phen-containing (pc-) (7 mM) 1.0 M NaOH electrolytes.

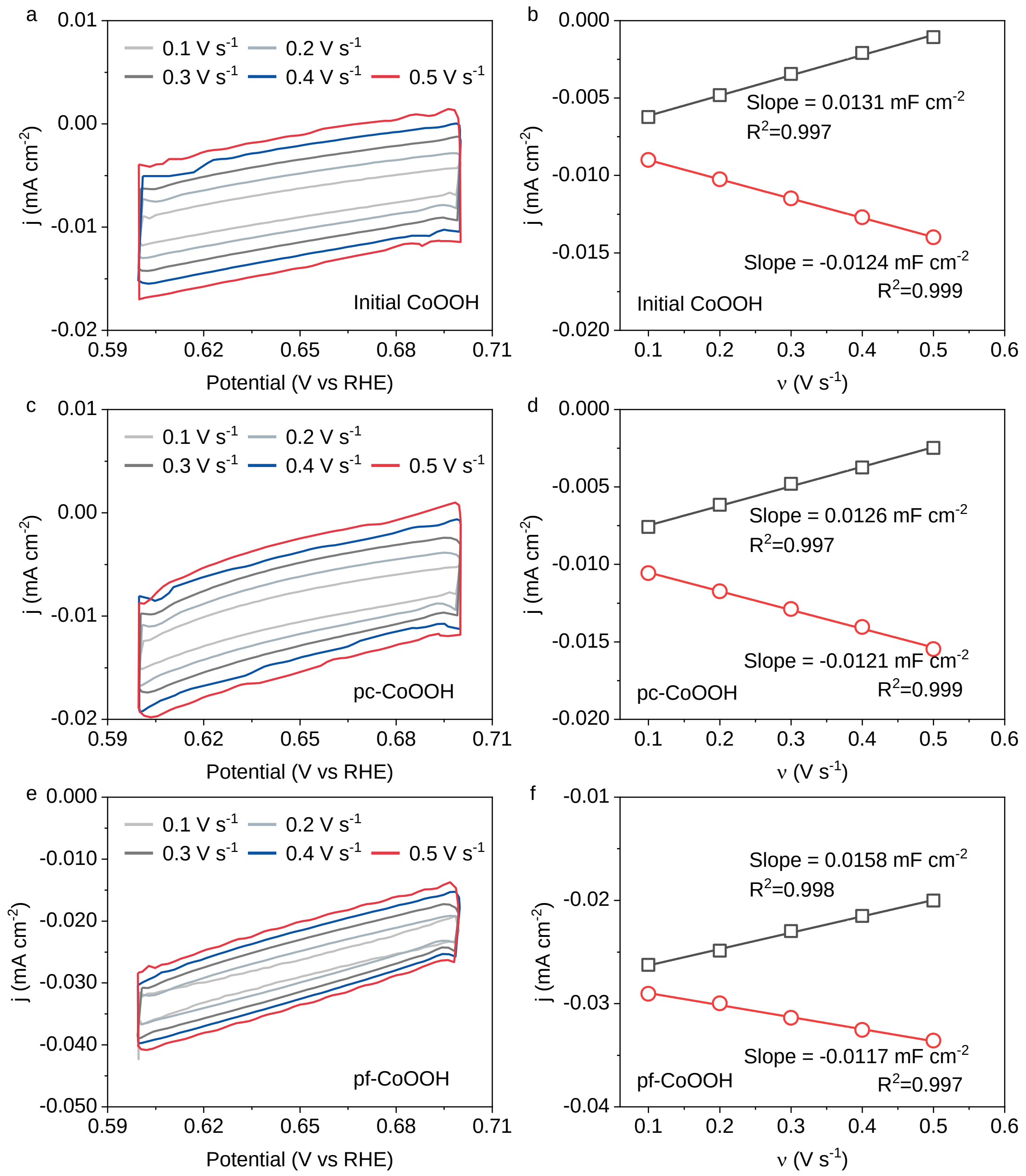

Supplementary Fig. 2 | Double-layer capacitance measurements. CV curves in 1.0 M NaoH of **(a)** pristine CoOOH, **(c)** pc-CoOOH, and **(e)** pf-CoOOH in a non-Faradic region (0.6 ~ 0.7 V) with a scan rate of 0.1 V s^{-1} to 0.5 V s^{-1}. Liner fitting of the cathodic and anodic charging current density at 0.65 V_{RHE} of **(b)** pristine CoOOH, **(d)** pc-CoOOH, and **(f)** pf-CoOOH as a function of scan rate. The pf-CoOOH and pc-CoOOH on FTO were obtained after 10 h chronoamperometry treatments of pristine CoOOH at 1.7 V_{RHE} in phen-free and phen-containing 1.0 M NaOH, respectively.

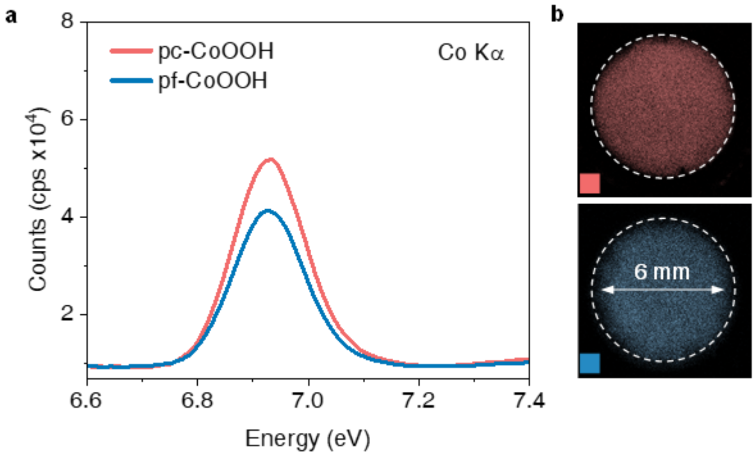

Supplementary Fig. 3 | XRF data. (a) The counts of Co K_{α} emission and **(b)** the corresponding Co K_{α} mapping of pc-CoOOH and pf-CoOOH on FTO electrodes by XRF. The pf-CoOOH and pc-CoOOH on FTO were obtained after 10 h chronoamperometry treatments of pristine CoOOH at 1.7 V_{RHE} in phen-free and phen-containing 1.0 M NaOH, respectively.

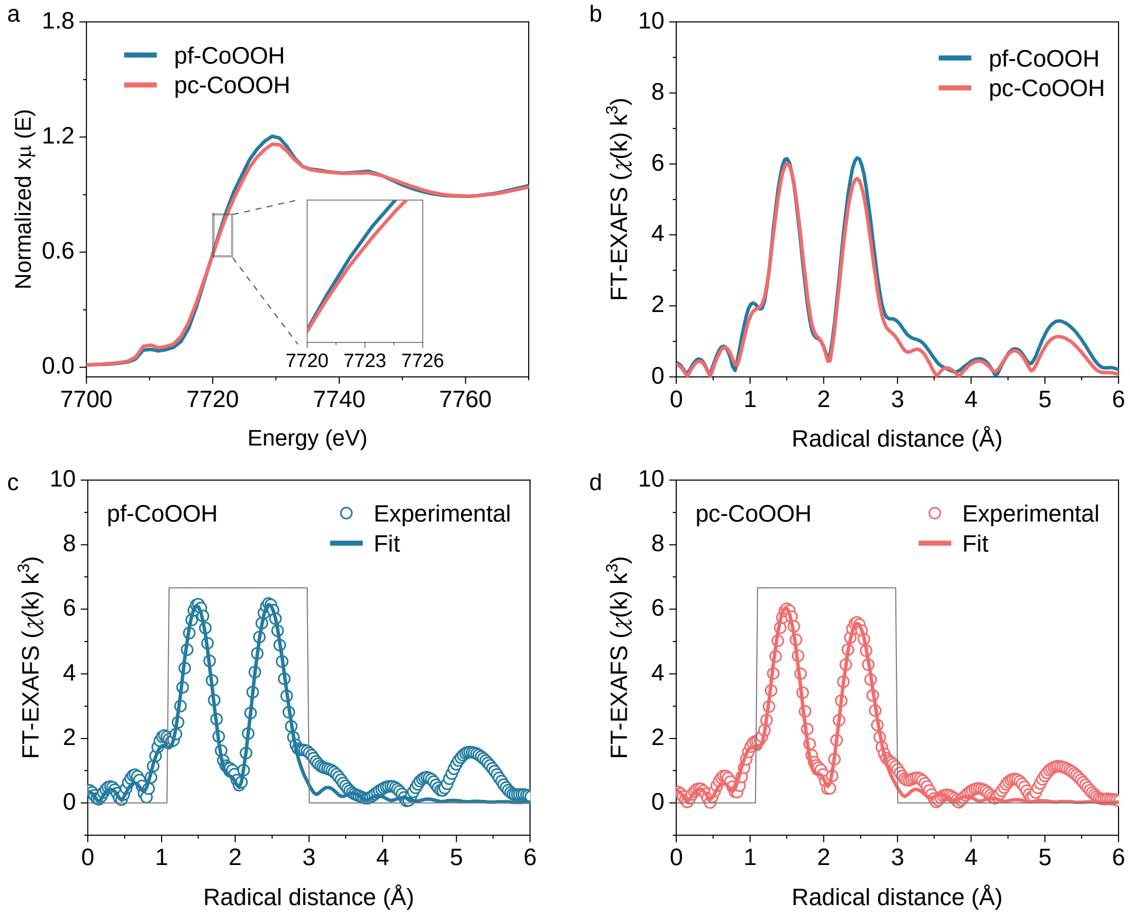

Supplementary Fig. 4 | XAFS data. (a) Co K-edge XANES spectra of as-prepared pf-CoOOH and pc-CoOOH, and **(b)** the corresponding k^3-weighted Fourier-transformed EXAFS. **(c)** Fitting curves of Co K-edge EXAFS in R spaces for pf-CoOOH and **(d)** for pc-CoOOH. The pf-CoOOH and pc-CoOOH were obtained after 10 h chronoamperometry treatments of pristine CoOOH at 1.7 V_{RHE} in phen-free and phen-containing 1.0 M NaOH, respectively.

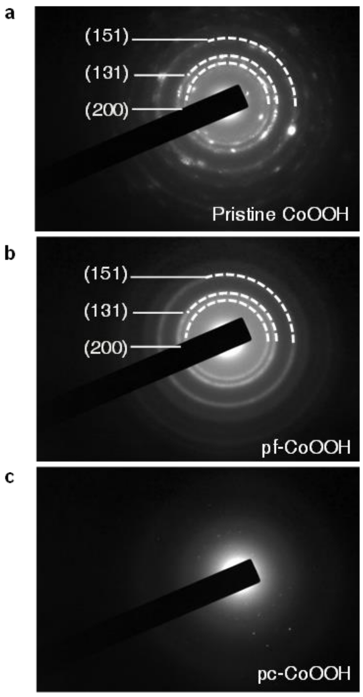

Supplementary Fig. 5 | SAED images. (a) Pristine CoOOH. **(b)** pf-CoOOH. **(c)** pc-CoOOH. The pf-CoOOH and pc-CoOOH were obtained after 10 h chronoamperometry treatments of pristine CoOOH at 1.7 V_{RHE} in phen-free and phen-containing 1.0 M NaOH, respectively.

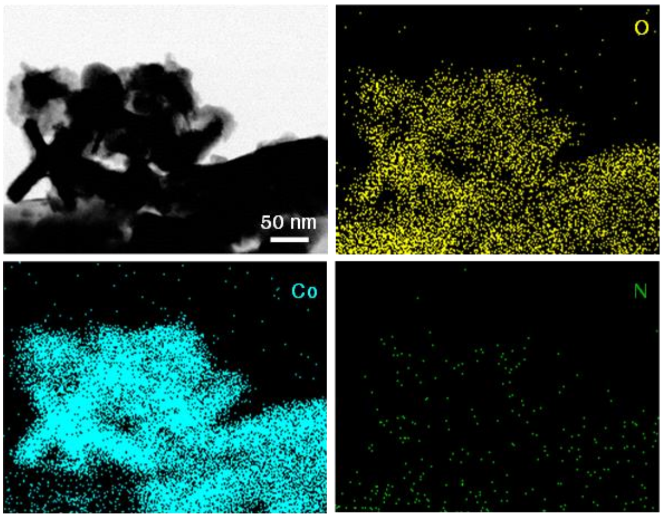

Supplementary Fig. 6 | TEM image and element mapping. The TEM and element mapping of pc-CoOOH. The pc-CoOOH was obtained after 10 h chronoamperometry treatments of pristine CoOOH at 1.7 V_{RHE} in phen-containing 1.0 M NaOH.

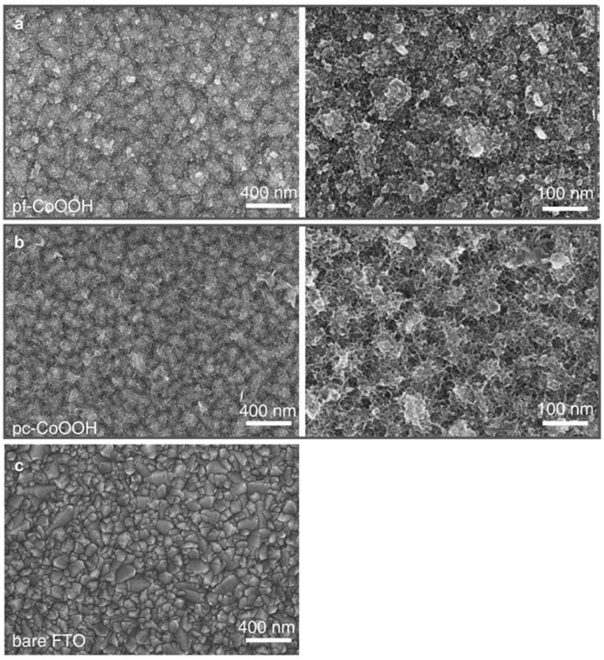

Supplementary Fig. 7 | SEM images. The morphology of **(a)** pf-CoOOH and **(b)** pc-CoOOH. The pf-CoOOH and pc-CoOOH were obtained after 10 h chronoamperometry treatments of pristine CoOOH at 1.7 V_{RHE} in phen-free and phen-containing 1.0 M NaOH, respectively. **(c)** bare FTO. The pf-CoOOH and pc-CoOOH films demonstrate a slight difference in surface texture.

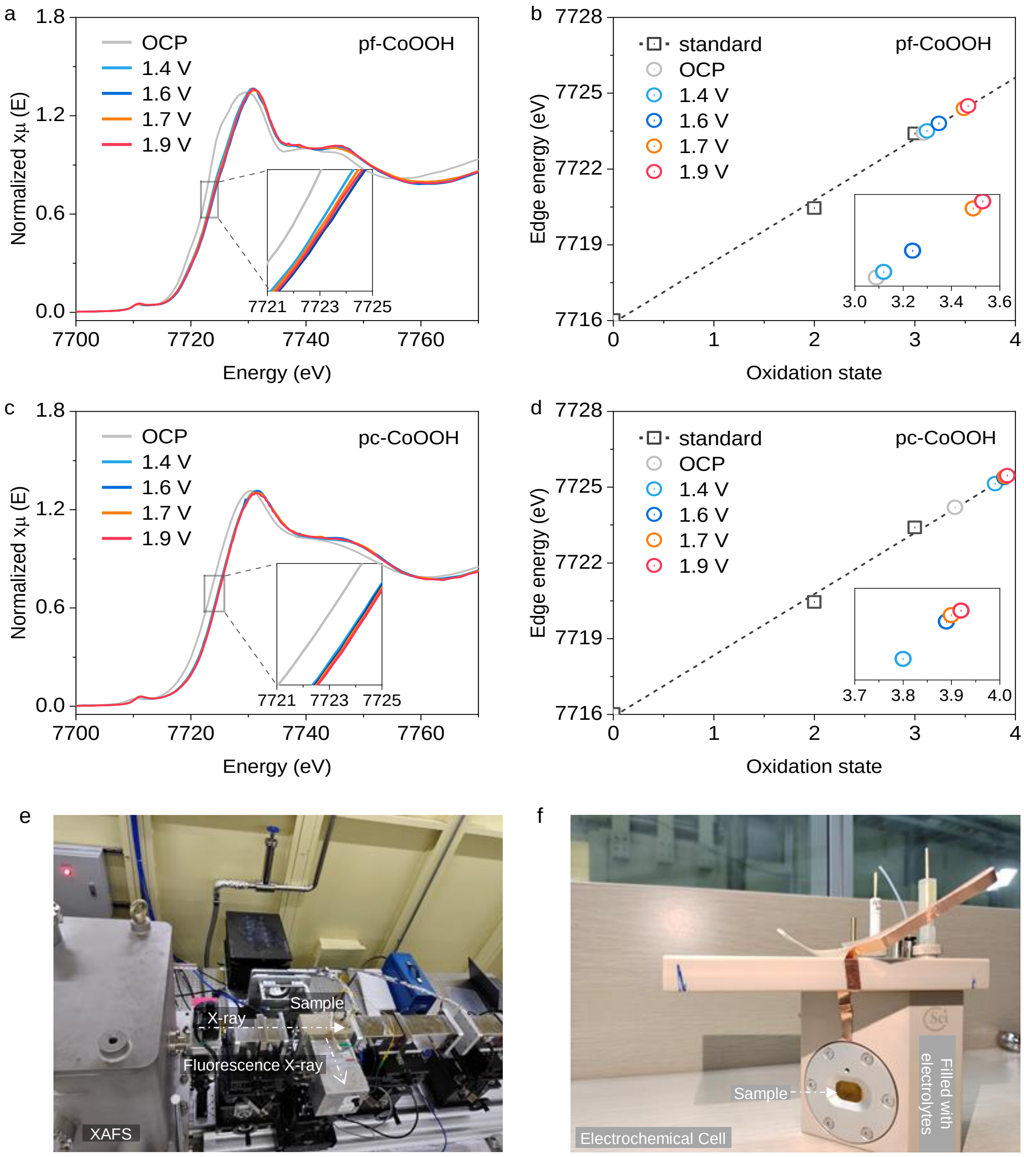

Supplementary Fig. 8 | In-situ XAFS data. (a) Co K-edge XANES of pf-CoOOH different potentials. **(b)** The corresponding linear fit of Co oxidation state for pf-CoOOH. **(c)** Co K-edge XANES of pc-CoOOH different potentials. **(d)** The corresponding linear fit of Co oxidation state for pc-CoOOH. The pf-CoOOH and pc-CoOOH on carbon paper were obtained after 10 h chronoamperometry treatments of pristine CoOOH at 1.7 V_{RHE} in phen-free and phen-containing 1.0 M NaOH, respectively. The images of (**e**) the in-situ XAFS equipment and (**f**) the electrochemical cell. Reference spectra include those of Co foil, CoO, and Co_2O_3 in Supplementary Fig. 9.

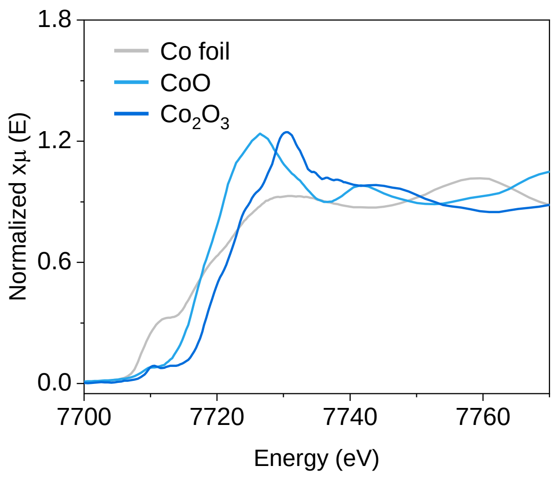

Supplementary Fig. 9 | XAFS data. Co K-edge XANES of Co foil, CoO, and Co_2O_3.

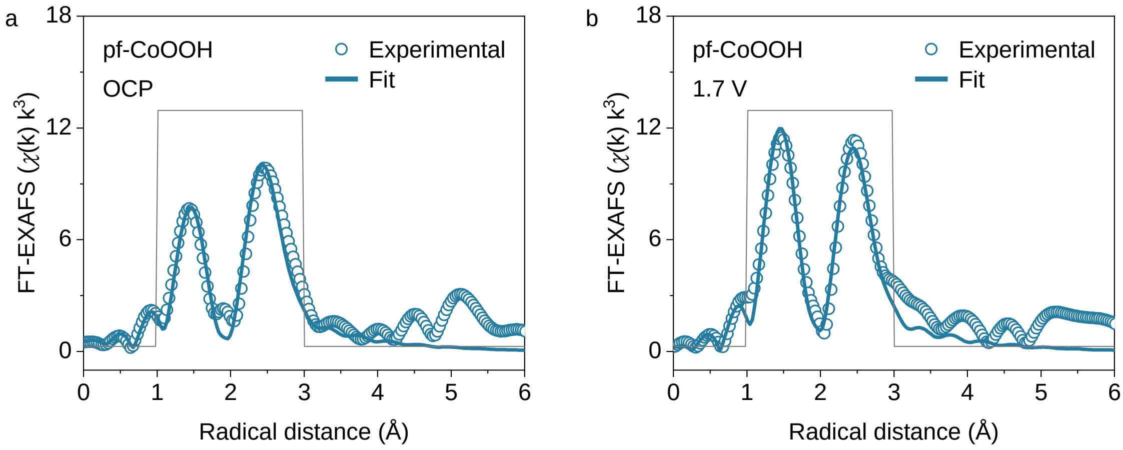

Supplementary Fig. 10 | Fitting curves of Fourier transformed k^3-weighted Co K-edge EXAFS in R spaces for pf-CoOOH. Fitting curves of Co K-edge EXAFS in R space for pf-CoOOH **(a)** at OCP and **(b)** at 1.7 V_{RHE}. The pf-CoOOH on carbon paper was obtained after 10 h chronoamperometry treatments of pristine CoOOH at 1.7 V_{RHE} in phen-free 1.0 M NaOH.

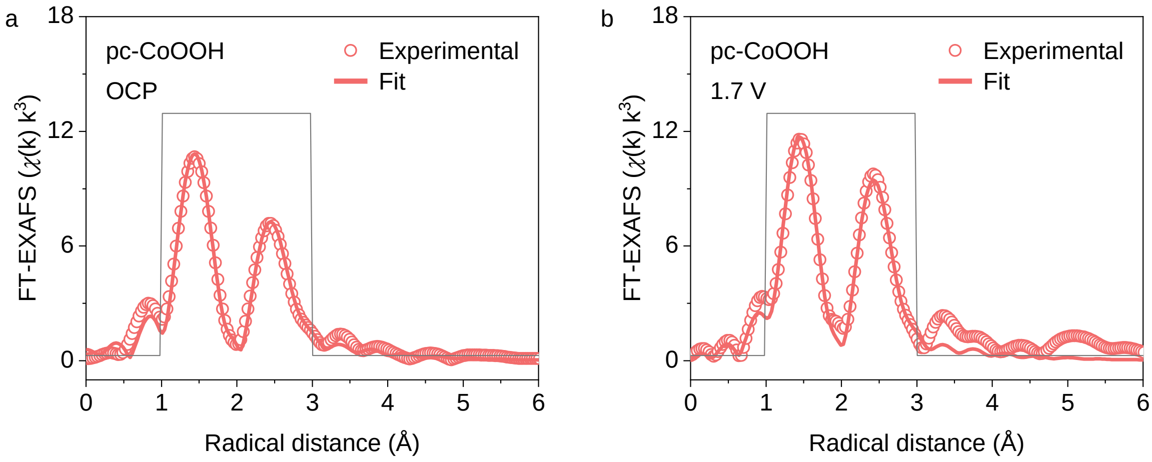

Supplementary Fig. 11 | Fitting curves of Fourier transformed k^3-weighted Co K-edge EXAFS in R spaces for pc-CoOOH. Fitting curves of Co K-edge EXAFS in R space for pc-CoOOH **(a)** at OCP and **(b)** at 1.7 V_{RHE}. The pc-CoOOH on carbon paper was obtained after 10 h chronoamperometry treatments of pristine CoOOH at 1.7 V_{RHE} in phen-containing 1.0 M NaOH.

Supplementary Table 1 | The best-fitting parameters for FT-EXAFS in R space of pf-CoOOH and pc-CoOOH.

Sample Name	/	Path	C.N.	σ^2 (10^{-3} Å^2)	R (Å)	ΔE_0 (eV)	R-factor (%)
pf-CoOOH	As-prepared	Co-O	3.84±0.32	3.01±1.01	1.930	-0.11±0.88	0.63
		Co-Co	3.30±0.51	4.45±1.18	2.887		
	OCP	Co-O	3.82±0.12	2.33±0.38	1.909	-2.79±2.66	3.09
		Co-Co	3.20±0.15	1.15±1.23	2.825		
	1.7 V_{RHE}	Co-O	4.95±0.69	0.76±1.6	1.892	-2.47±1.43	0.91
		Co-Co	4.00±0.02	2.26±0.79	2.832		
pc-CoOOH	As-prepared	Co-O	3.62±0.27	2.76±0.91	1.930	0.67±0.79	0.56
		Co-Co	2.99±0.46	4.55±1.18	2.887		
	OCP	Co-O	5.11±0.07	1.16±0.60	1.893	-2.31±1.08	0.47
		Co-Co	4.07±1.12	5.51±2.32	2.826		
	1.7 V_{RHE}	Co-O	6.05±0.57	2.26±1.11	1.882	-3.53±1.00	0.50
		Co-Co	4.99±0.18	4.88±0.70	2.831		

C.N.: Coordination number; R: interatomic distance; σ^2: disorder factors; ΔE_0: energy shifts**.** The amplitude factor S_0^2 was determined by fitting standard Co foil with a fixed coordination number based on the known crystal structure.

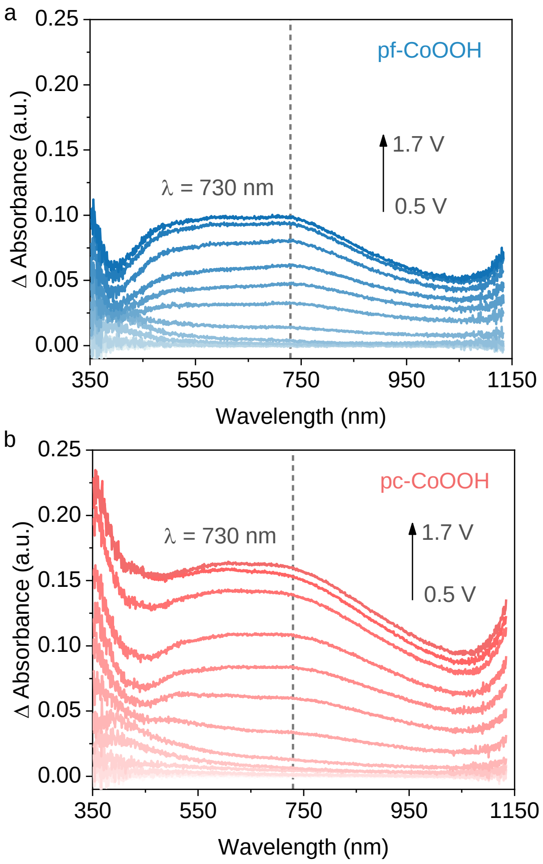

Supplementary Fig. 12 | In-situ UV-Vis. Potential-dependent UV-Vis spectra of **(a)** pf-CoOOH and **(b)** pc-CoOOH were recorded from 0.5 to 1.7 V_{RHE} in 1.0 M NaOH. The background was deducted at 0.5 V_{RHE}. Before the UV-Vis measurements, the films were obtained after 10 h chronoamperometry treatments of pristine CoOOH at 1.7 V_{RHE} in phen-free and phen-containing (7 mM) 1.0 M NaOH.

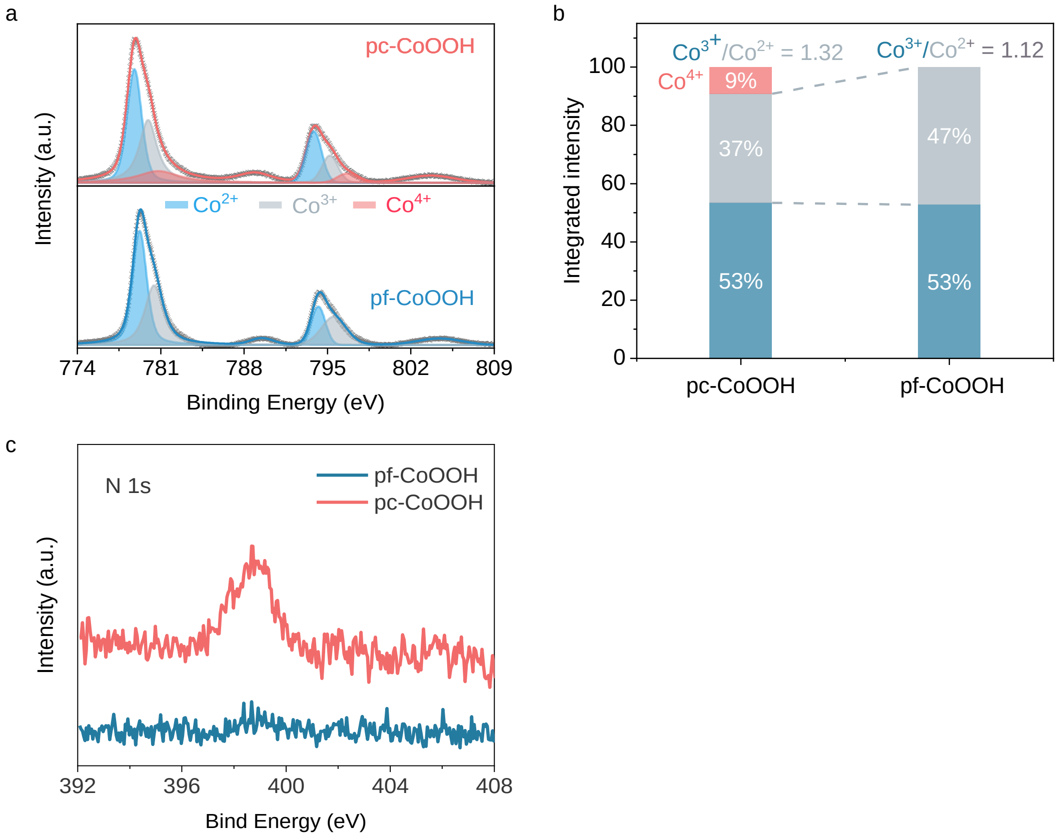

Supplementary Fig. 13 | XPS of pf-CoOOH and pc-CoOOH. (a) Co 2*p* XPS spectra of pf-CoOOH and pc-CoOOH. **(b)** The ratio of Co^{3+}/Co^{2+} and Co^{4+} in pf-CoOOH and pc-CoOOH is determined by the area of the respective peak after Co 2*p* fitting. **(c)** The corresponding N 1*s* XPS. The pf-CoOOH and pc-CoOOH on carbon paper were obtained after 10 h chronoamperometry treatments of pristine CoOOH at 1.7 V_{RHE} in phen-free and phen-containing 1.0 M NaOH, respectively.

Supplementary Table 2 | The composition of pc-CoOOH analyzed by XPS. The pc-CoOOH on FTO was obtained after 10 h chronoamperometry treatments of pristine CoOOH at 1.7 V_{RHE} phen-containing 1.0 M NaOH.

	**Co 2*p***	**N 1*s***	**Co/N**
pc-CoOOH	89.47%	10.53%	8.49

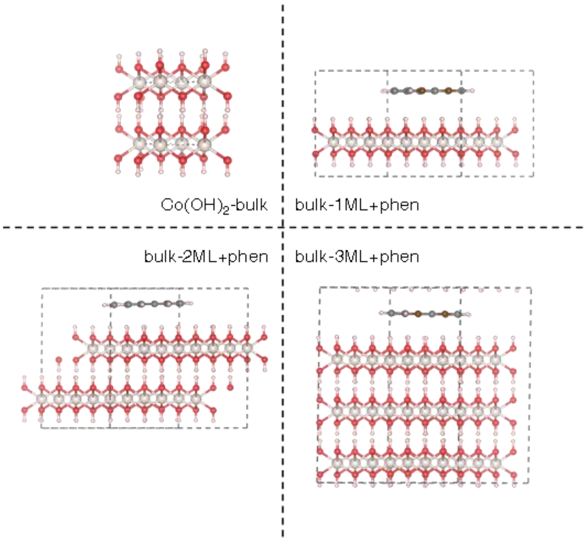

Supplementary Fig. 14 | DFT models. The structure schemes of phen-free $Co(OH)_2$, high(H)-, middle(M)-, and low(L)-density of phen (i.e., 1 ML, 0.5 ML, and 0.33 ML phen) embedded $Co(OH)_2$. ML is the abbreviation of a monolayer.

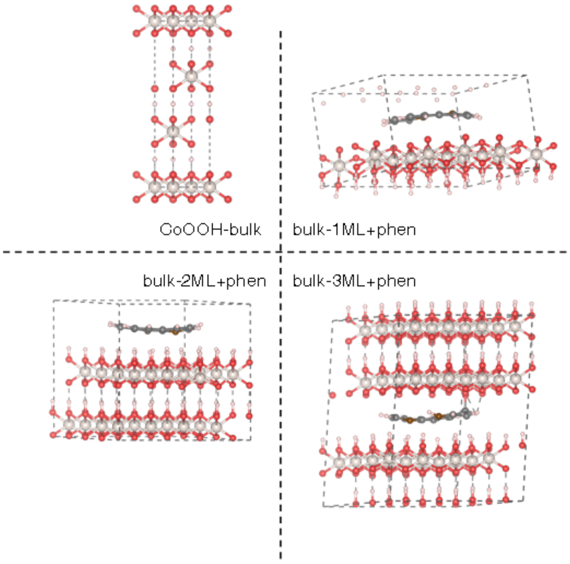

Supplementary Fig. 15 | DFT models. The structure schemes of phen-free CoOOH, high(H)-, middle(M)-, and low(L)-density of phen (i.e., 1 ML, 0.5 ML, and 0.33 ML phen) embedded CoOOH. ML is the abbreviation of a monolayer.

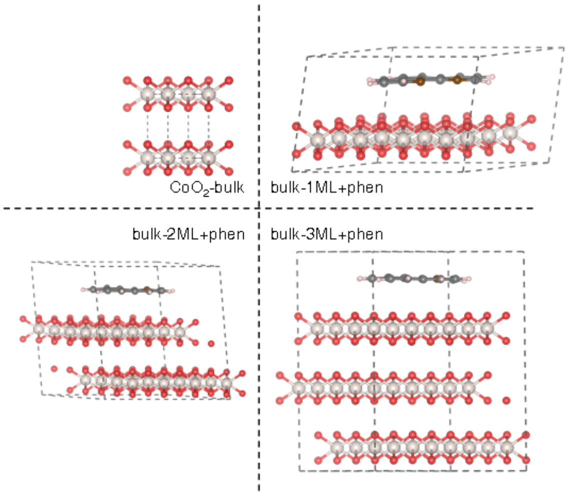

Supplementary Fig. 16 | DFT models. The structure schemes of phen-free CoO_2, high(H)-, middle(M)-, and low(L)-density of phen (i.e., 1 ML, 0.5 ML, and 0.33 ML phen) embedded CoO_2. ML is the abbreviation of a monolayer.

Supplementary Table 3 | Thermodynamic correction used in the free energy calculations. Zero-point energies are calculated with experimental vibrational data in reference[1,2], and the integrated heat capacity ($\delta H^{0\sim298K}$) and entropy at 298.15 K are obtained from reference[3]. For water, the entropy is calculated at 0.035 bar through $S = S_0 + k_B T ln\,(p\,/\,p0)$ to derive the chemical potential of liquid water, because at this pressure gas-phase water is in equilibrium with liquid water at 298.15 K. The solvation energies (E_{solv}) are evaluated from AIMD simulation by filling the vacuum with liquid water with a thickness that is equivalent to 5 water bilayers. It is worth noting that, in comparison with our previous work[4-6], the solvation energy of surface OH for Co-based hydroxides compounds is about -0.1 eV smaller, because of high OH density in the current case which leads to a very limited amount of hydrogen bonds formed with interface water. And the solvation energy of OH for molecules is about -0.6 eV.

	ZPE (eV)	δ_H@298K(eV)	TS@298K(eV)	E_{solv}(eV)
H_2O(g)	0.56	0.10	0.68	
H_2	0.27	0.09	0.40	
(O)-H* from oxides	0.30	0.01	0.01	
(O)H	0.32	0.00	-0.02	-0.10/-0.60

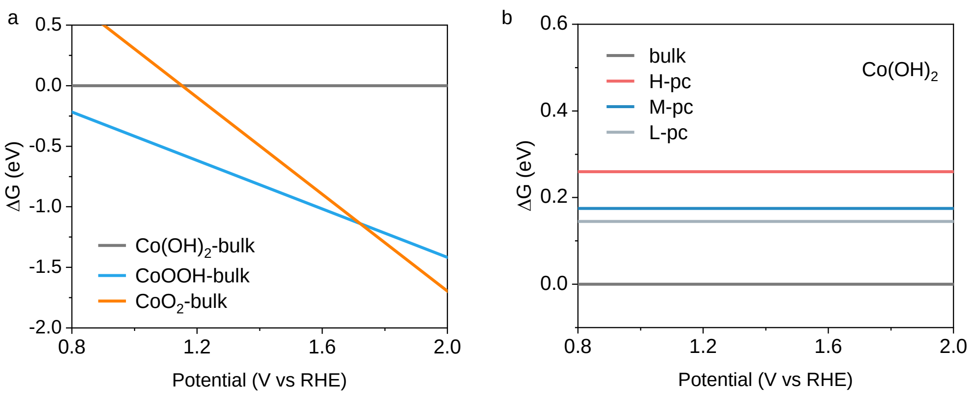

Supplementary Fig. 17 | The bulk free energy diagrams. (a) The bulk free energy diagrams of $Co(OH)_2$, CoOOH, and CoO_2. **(b)** The bulk free energy diagrams of layered $Co(OH)_2$ contain high-, middle-, and low-density of phen that are inserted into interlayers. In the case of $Co(OH)_2$, phen can coordinate with Co^{2+}, thus the insertion of phen into $Co(OH)_2$ only be considered theoretically.

Supplementary Table 4 | Formation energy of phen-containing bulk CoOOH and CoO_2 with different numbers of monolayers.

Formation energy (eV/Co)	CoOOH+phen	CoO_2+phen
1 ML-bulk + phen (H-pc)	0.63	-0.07
2 ML-bulk + phen (M-pc)	0.40	-0.03
3 ML-bulk + phen (L-pc)	0.27	-0.01

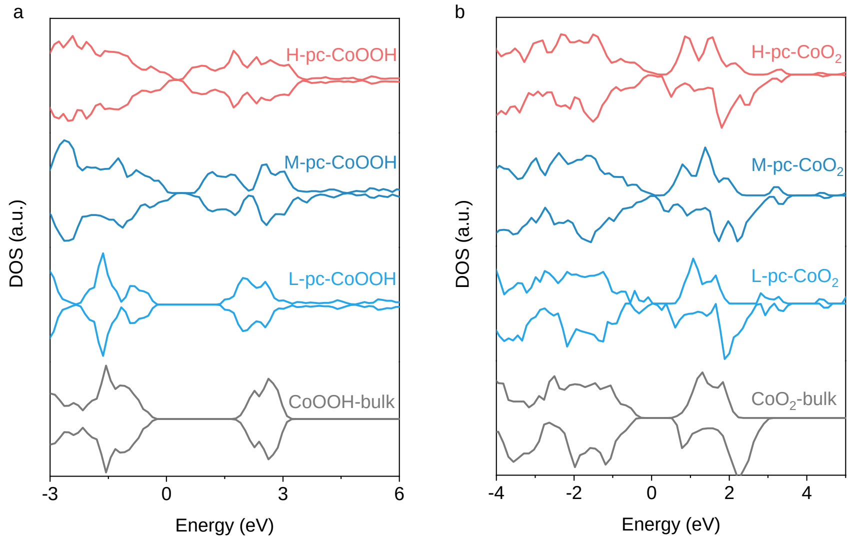

Supplementary Fig. 18 | The densities of states. The densities of states (DOS) of phen-free or high-density, middle-density, and low-density phen-containing bulk **(a)** CoOOH and **(b)** CoO_2.

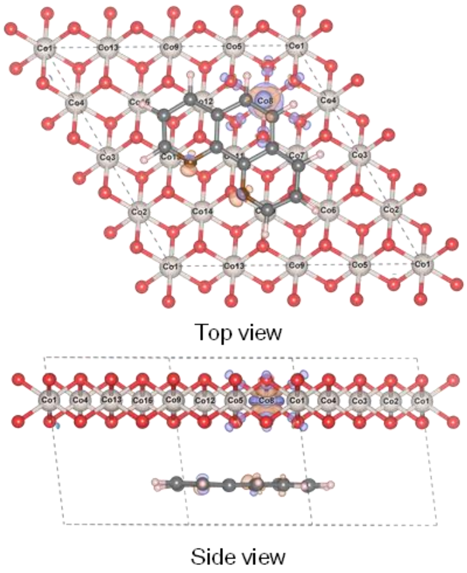

Supplementary Fig. 19 | Charge density differences of H-pc-CoO_2. The purple isosurfaces correspond to charge densities of 0.011 e/Bohr^3 and represent an increase in the total charge density. The orange isosurfaces correspond to charge densities of -0.011 e/Bohr^3 and represent a decrease in the total charge density.

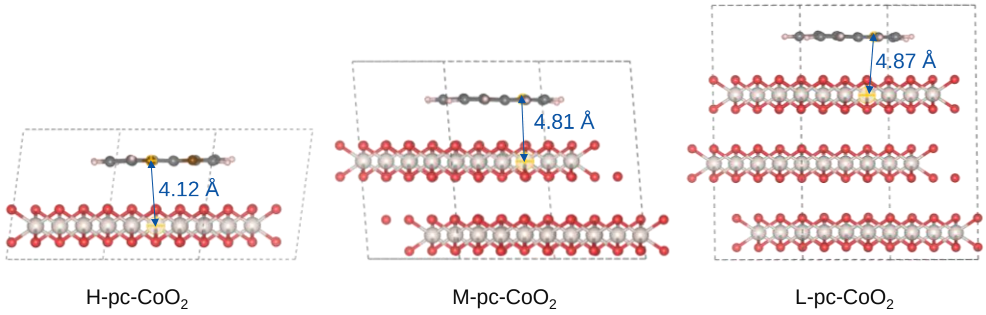

Supplementary Fig. 20 | The geometric structures of layered CoO_2 containing high(H)-, middle(M)-, and low(L)-density of phen (i.e., 1 ML, 0.5 ML, and 0.33 ML phen) in the interlayers. The dark blue notes indicate the shortest distance between the N atom and the Co atom.

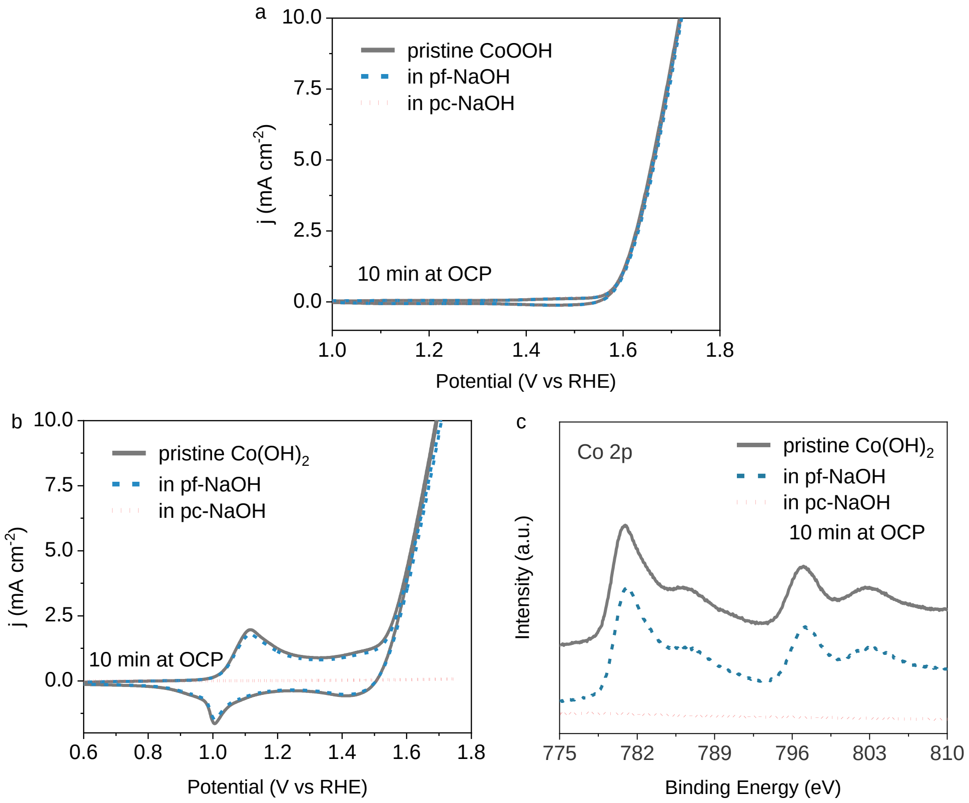

Supplementary Fig. 21 | The coordination ability of Co^{2+} and Co^{3+} with phen on electrodes. **(a)** The CV curves of the CoOOH catalysts on FTO in 1.0 M NaOH after holding the potential at OCP in phen-free and phen-containing 1.0 M NaOH electrolytes, respectively. **(b)** The CV curves of the $Co(OH)_2$ catalysts on FTO in 1.0 M NaOH after holding the potential at OCP in phen-free and phen-containing 1.0 M NaOH electrolytes, respectively, and **(c)** the corresponding Co 2*p* XPS. The concentration of phen is 7 mM.

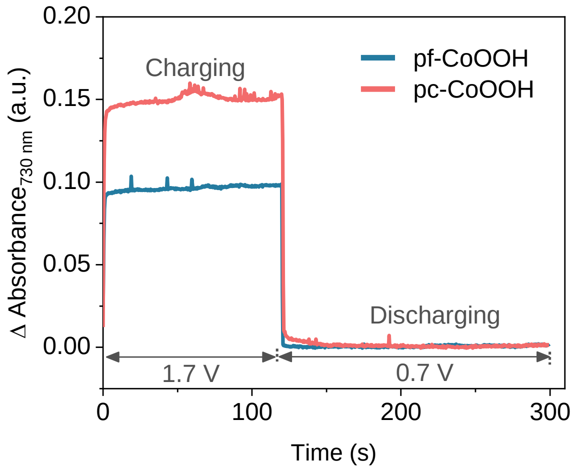

Supplementary Fig. 22 | In-situ UV-Vis under charging and discharging. The absorbance difference of pf-CoOOH and pc-CoOOH at 730 nm upon charging at 1.7 V_{RHE} and discharging at 0.7 V_{RHE} in 1.0 M NaOH. The background was deducted at 0.5 V_{RHE}. The absorbance of pf-CoOOH and pc-CoOOH decreases to zero at 0.7 V_{RHE}, indicating the different amounts of oxidative charges stored. The pf-CoOOH and pc-CoOOH on FTO were obtained after 10 h chronoamperometry treatments of pristine CoOOH at 1.7 V_{RHE} in phen-free and phen-containing 1.0 M NaOH, respectively.

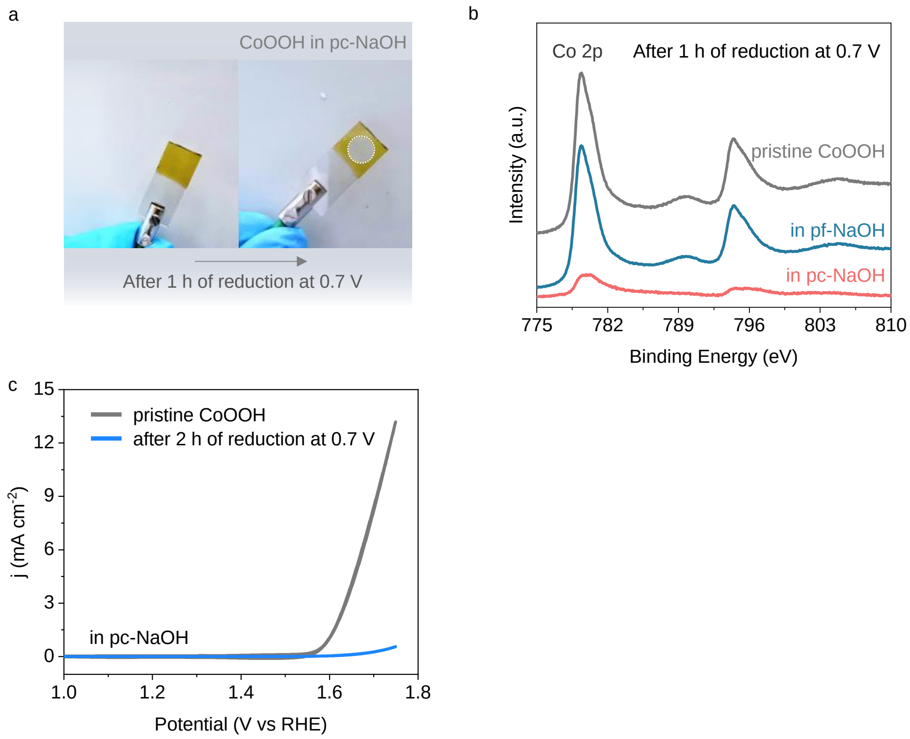

Supplementary Fig. 23 | The reduction of Co^{3+} to Co^{2+} leads to the formation of soluble Co-phen species. (a) The images of pristine CoOOH and CoOOH on FTO after 1.0 h of reduction at 0.7 V_{RHE} in phen-containing 1.0 M NaOH, **(b)** and their corresponding Co 2*p* XPS. **(c)** The CV curves of CoOOH after 2.0 h of reduction at 0.7 V in phen-containing NaOH. The phen concentration is 7 mM in all cases.

Supplementary discussion to Supplementary Figs. 20-23

Based on the valence-dependent deposition/dissolution experiments, we can draw the following conclusions:

(1) Reduction of CoOOH at 0.7 V_{RHE} leads to the formation of Co^{2+} that coordinates with phen to generate soluble Co-phen species in the phen-containing electrolyte. Discharging the Co^{3+} sites and reducing them to Co^{2+} after switching the potential from 1.7 to 0.7 V_{RHE} (that is, close to the offset potential of $Co^{3+/2+}$, as shown in Fig. 2c in the *main text*) in phen-free 1.0 M NaOH electrolyte leads to the reduction of Co sites to the similar level (Supplementary Fig. 22). Holding the potential at 0.7 V_{RHE} around 2.0 h results in a complete dissolution of the CoOOH catalyst thereby losing OER activity in phen-containing NaOH (Supplementary Fig. 23). To

elucidate the coordination of Co^{2+} sites with phen, we synthesized $Co^{II}(OH)_2$ film that coordinated with free phen in electrolytes dissolving into the electrolyte at OCP within only 10 min (Supplementary Figs. 21b-21c). These results demonstrate a valence-dependent deposition/dissolution in the Co-phen system.

(2) The Co^{3+} sites stay on the electrode in the phen-containing electrolyte at OCP. As shown in Supplementary Fig. 21a, the pristine $Co^{III}OOH$ stays on the electrode in the phen-containing 1.0 M NaOH electrolyte over 10 min at OCP, and its activity remains relative to the $Co^{III}OOH$ in the phen-free electrolyte.

(3) The non-covalent interaction between CoO_2 and phen.

(i) The results present that phen can induce and stabilize the high-valence Co^{4+} (Fig. 1, Fig. 2, and Supplementary Fig. 1), and improve the charge transfer of the semiconducting CoO_2 phase. The interplay between phen with CoO_2 is significantly different from that of phen and $Co(OH)_2$ or CoOOH. DFT calculations further reveal that phen cannot coordinate with Co^{4+} but works through forming a non-covalent interaction.

(ii) The covalent and non-covalent interactions can be distinguished by the stabilization energy and distance in equilibrium. The interactions with low stabilization energies and relatively long distances are ascribed to the "non-covalent interactions"[7]. Typically, covalent interactions are short-range and are generally shorter than 2 Å, and non-covalent interactions are known to act at distances >2 Å[8]. In this work, the shortest distance between the N atom and Co atom is 4.12 Å in H-pc-CoO_2 (Supplementary Fig. 20). Moreover, the calculated interaction energies within the range of 64~113 kJ $mol_{(phen)}^{-1}$, depending on low(L)-, middle(M)-, and high(H)-density phen, which are considerably smaller than the stabilization energy of covalent bond of about 200~300 kJ mol^{-1}. Thus, the interaction between phen with CoO_2 has been considered non-covalent. This model of H-density phen is close to the phen content in the pc-CoOOH catalysts.

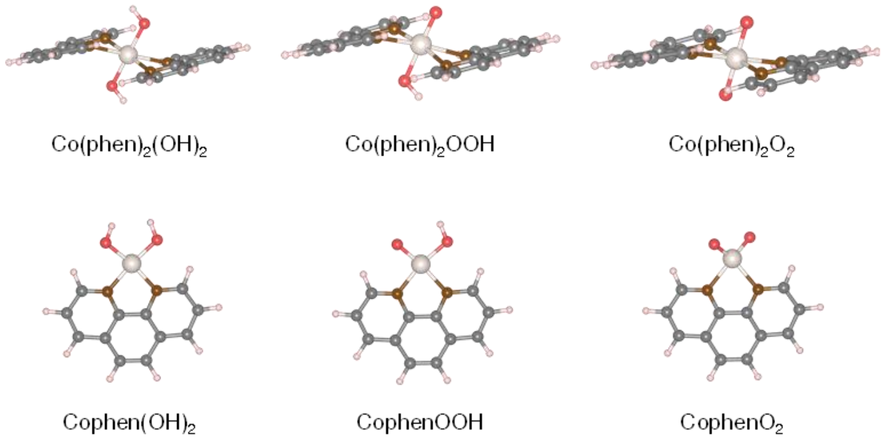

Supplementary Fig. 24 | The phen-coordinated molecule geometric structures. The DFT models of Co(phen)$_2$(OH)$_2$, Co(phen)$_2$OOH, Co(phen)$_2$O$_2$, Cophen(OH)$_2$, CophenOOH and CophenO$_2$. The energy-unfavorable structures have been discussed in the main text.

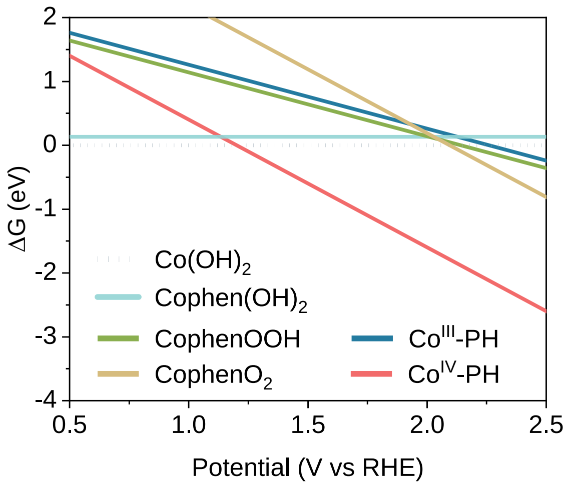

Supplementary Fig. 25 | The free energy diagrams. The free energy diagrams of $Co(OH)_2$, Cophen$(OH)_2$, CophenOOH, CophenO_2, phen-containing layered CoOOH (Co^{III}-PH), and CoO_2 (Co^{IV}-PH) are based on DFT calculations.

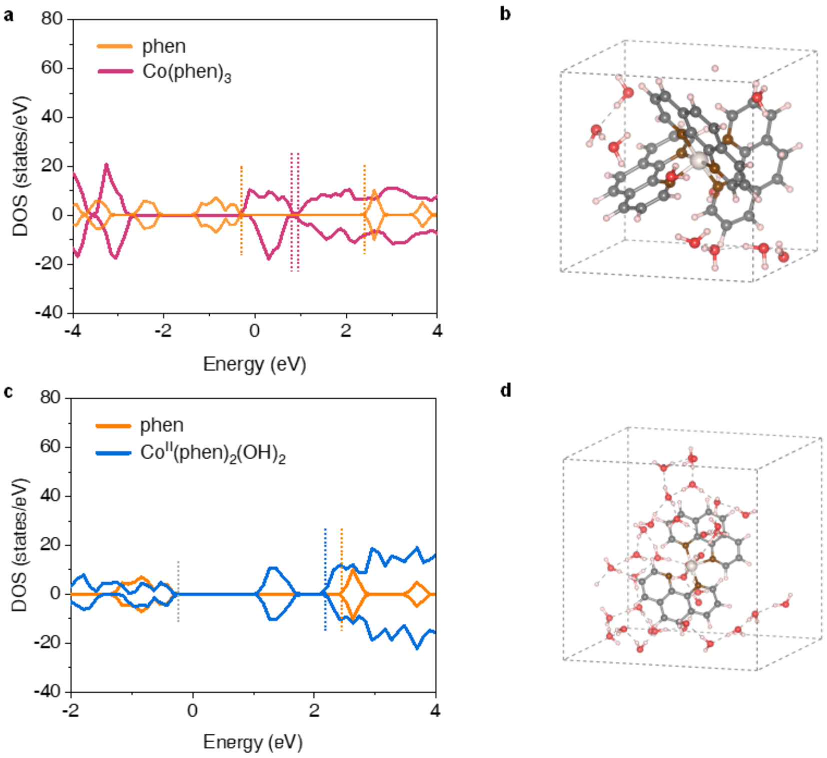

Supplementary Fig. 26 | The DOS and model of Co(phen)$_3$ and Co^{II}(phen)$_2$(OH)$_2$. (a) The DOS and PDOS of Co(phen)$_3$ and phen. **(b)** The DFT model of Co(phen)$_3$ in water. **(c)** The DOS and PDOS of phen and Co^{II}(phen)$_2$(OH)$_2$. **(d)** The DFT model for Co^{II}(phen)$_2$(OH)$_2$ in water.

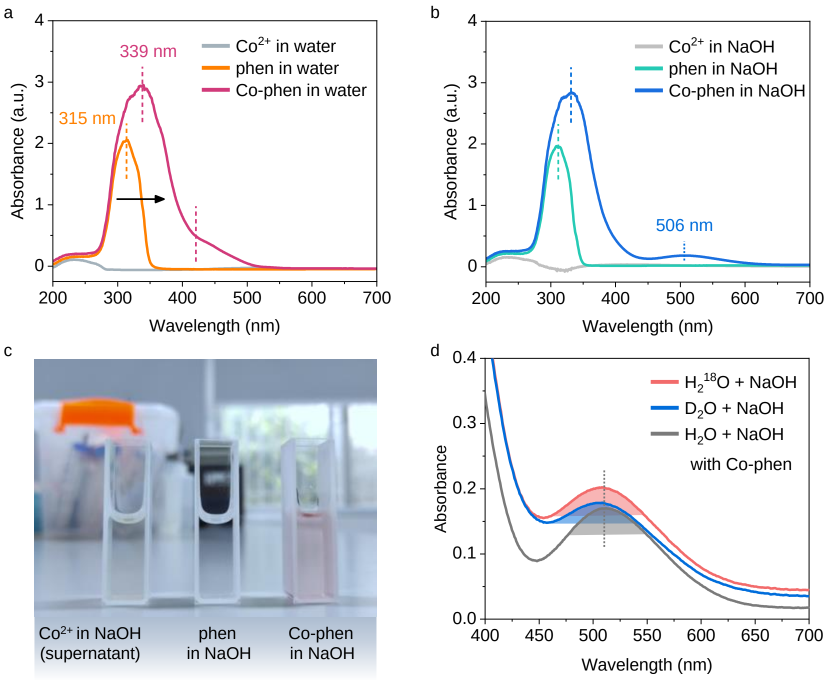

Supplementary Fig. 27 | The UV-Vis spectra. (a) The UV-Vis spectra of Co^{2+}, phen, and Co-phen in water and **(b)** 1.0 M NaOH, respectively. The absorption peak shows a slightly red-shift from 315 nm for phen to a higher wavelength at 339 nm for Co-phen in water, suggesting the coordination of Co^{2+} and phen. In addition, a new absorption band appears at approximately 450 nm, which is assigned to the ligand-to-metal charge transfer. In 1.0 M NaOH, this ligand-to-metal absorption peak redshifts to ~506 nm. **(c)** The photographs of Co^{2+}, phen, and Co-phen in 1.0 M NaOH. **(d)** The UV-Vis spectra of Co-phen in $H_2{}^{18}O$, $D_2{}^{16}O$, and $H_2{}^{16}O$ with 1.0 M $Na^{16}OH$, respectively. The peak at ~506 nm shows a sslight shift due to the isotope effect[9-11], indicating the water-associated coordination. The background is deducted by the bare quartz cuvettes.

Supplementary discussion to Supplementary Figs. 26-27

DFT calculations of DOS for the $Co(phen)_3$ and $Co(phen)_2(OH)_2$ were carried out. Coordination between Co and phen yielded a narrow energy gap (Supplementary Fig. 26a). Thus, the absorption peak at 450 nm in Supplementary Fig. 27a could be ascribed to the Co-N coordination. While, $Co(phen)_2(OH)_2$ shows an obvious decrease in energy gap (Supplementary Fig. 26b), consistent with the redshift of the absorption peak of Co-N coordination (Supplementary Fig. 27b).

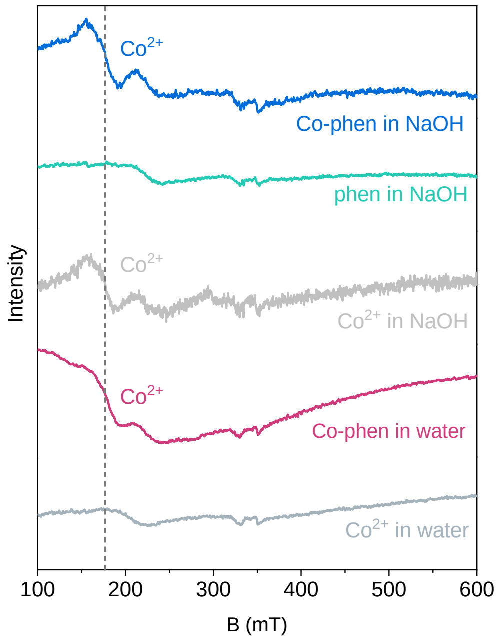

Supplementary Fig. 28 | The EPR spectra at 100 K. The EPR spectra of Co^{2+}, phen, and Co-phen in water and 1.0 M NaOH. The Co^{2+} in water is almost EPR silent. However, a broad paramagnetic signal[12,13] (g ≈ 4.31) appeared when phen was introduced, suggesting the coordination between phen and Co^{2+} induced a spin-state transition of Co^{2+}. In contrast to the situation in the water, a broad paramagnetic Co^{2+} signal was observed in NaOH electrolytes.

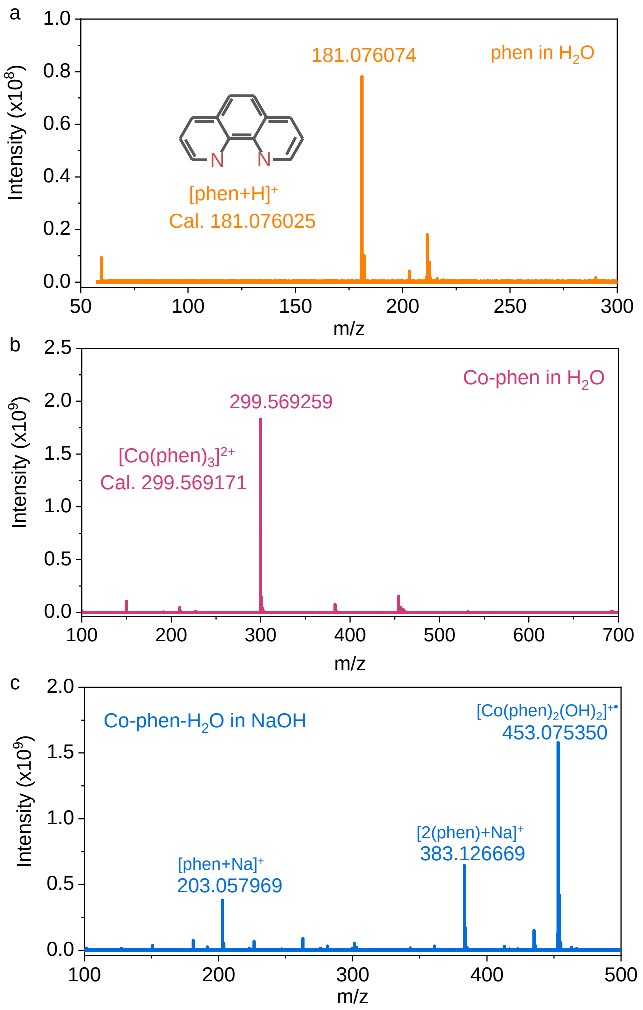

Supplementary Fig. 29 | The FT-ICR-MS spectra. (a) FT-ICR-MS spectra for phen in water, **(b)** Co-phen in water, and **(c)** Co-phen in 1.0 M NaOH. The spectra were recorded using dual electrospray ionization (ESI) source in the positive ion mode.

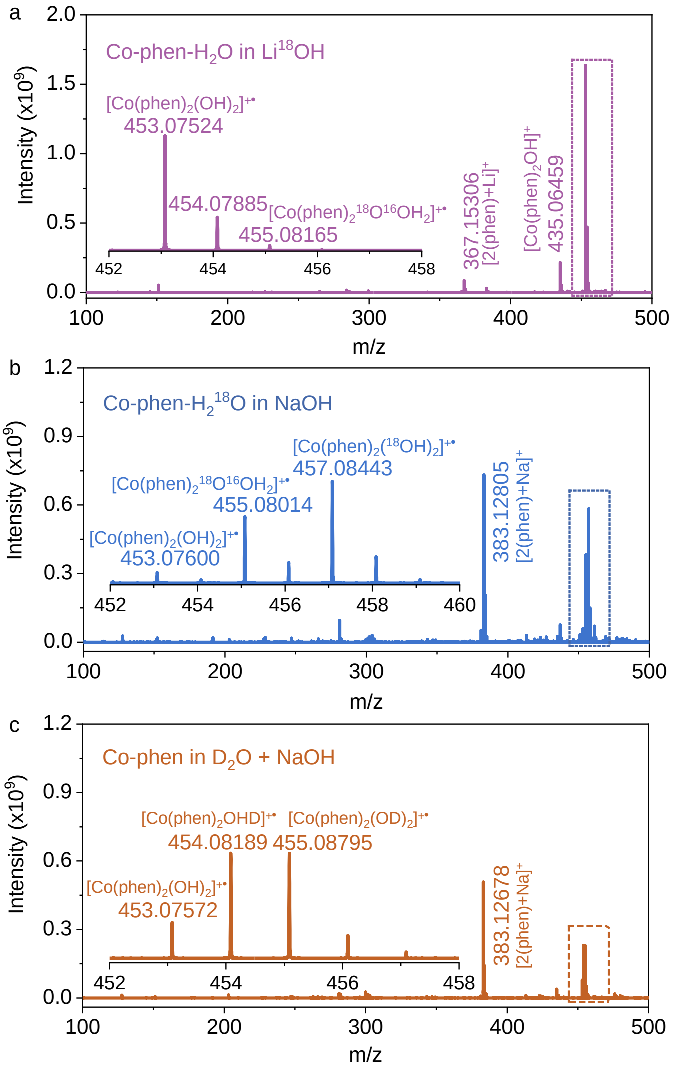

Supplementary Fig. 30 | Isotope-labeled FT-ICR-MS spectra. (a) FT-ICR-MS spectra for Co-phen in $H_2{}^{16}O$ with 1 M $Li^{18}OH$, **(b)** Co-phen in $H_2{}^{18}O$ (97.4%) with 1.0 M $Na^{16}OH$, and **(c)** Co-phen in D_2O (99.9%) with 1.0 M NaOH. The spectra were recorded using dual electrospray ionization (ESI) source in the positive ion mode.

Supplementary Table 5 | The molecular formula of isotope-labeled Co-phen in H_2O with 1.0 M NaOH or $Li^{18}OH$.

	Molecular formula	Experimental	Theoretical
**Co-phen in $H_2{}^{18}O$ with 1.0 M $Na^{16}OH$**	$[2phen+Na]^+$	383.12805	383.126169
	$[Co(phen)_2(^{18}OH)_2]^{+\bullet}$	457.084427	457.084115
	$[Co(phen)_2{}^{18}O^{16}OH)_2]^{+\bullet}$	455.080137	455.079869
	$[Co(phen)_2(^{16}OH)_2]^{+\bullet}$	453.076001	453.075622
	**Molecular formula**	**Experimental**	**Theoretical**
**Co-phen in $H_2{}^{16}O$ with 1.0 M $Li^{18}OH$**	$[2phen+Li]^+$	367.15306	367.0774
	$[Co(phen)_2(^{16}OH)_2]^{+\bullet}$	453.07524	453.075622
	$[Co(phen)_2{}^{18}O^{16}OH_2]^{+\bullet}$	455.081651	455.079869

Supplementary discussion to Supplementary Figs. 29-30 and Supplementary Table 5

We applied Fourier-transform ion cyclotron resonance mass spectrometry (FT-ICR-MS) to verify the molecular formula. The formula of Co-phen in water (Supplementary Fig. 29b) and 1.0 M NaOH (Supplementary Fig. 29c) by FT-ICR-MS are deduced to be dominated by $[Co(phen)_3]^{2+}$ species with an *m/z* ratio of 299.569 and $[Co(phen)_2(OH)_2]^{+\cdot}$ with an *m/z* ratio of 453.075, respectively. Furthermore, isotope-labeled $H_2{}^{18}O$, D_2O, and $Li^{18}OH$ are used to verify the source of OH of $[Co(phen)_2(OH)_2]^{+\cdot}$. As shown in Supplementary Figs. 30a-30b and Supplementary Table 5, the strongest peak at *m/z* 457.084 is ascribed to the $[Co(phen)_2(^{18}OH)_2]^{+\cdot}$ in 1.0 M $Na^{16}OH$-containing $H_2{}^{18}O$ electrolyte. In contrast, the strongest peak at *m/z* 453.075 is assigned to $[Co(phen)_2(^{16}OH)_2]^{+\cdot}$ in 1.0 M $Li^{18}OH$ containing H_2O electrolyte. Further, the peak at *m/z* 455.088 is assigned to $[Co(phen)_2(OD)_2]^{+\cdot}$ in 1.0 M NaOH D_2O electrolyte (Supplementary Fig. 30c). The results suggest that the OH^- in $[Co(phen)_2(OH)_2]^{+\cdot}$ is from H_2O.

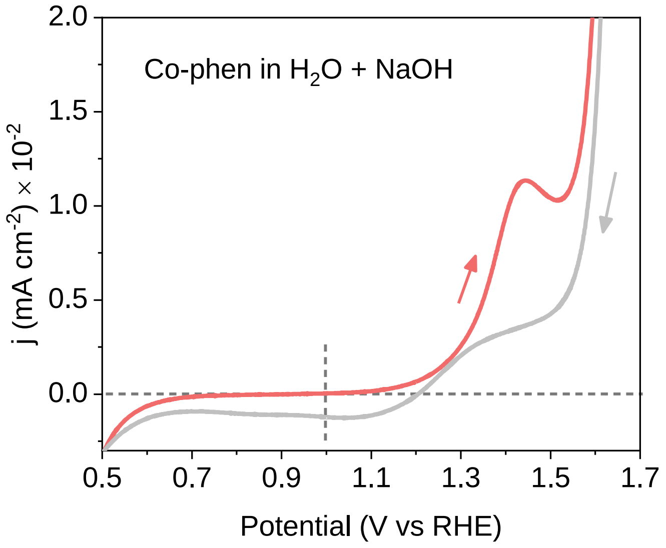

Supplementary Fig. 31 | The first CV of bare FTO in Co(phen)$_2$(OH)$_2$-containing 1.0 M NaOH. The red curve shows the oxidation process of Co^{2+}, and the grey one shows the reduction process of Co^{3+}/Co^{4+} to Co^{2+}.

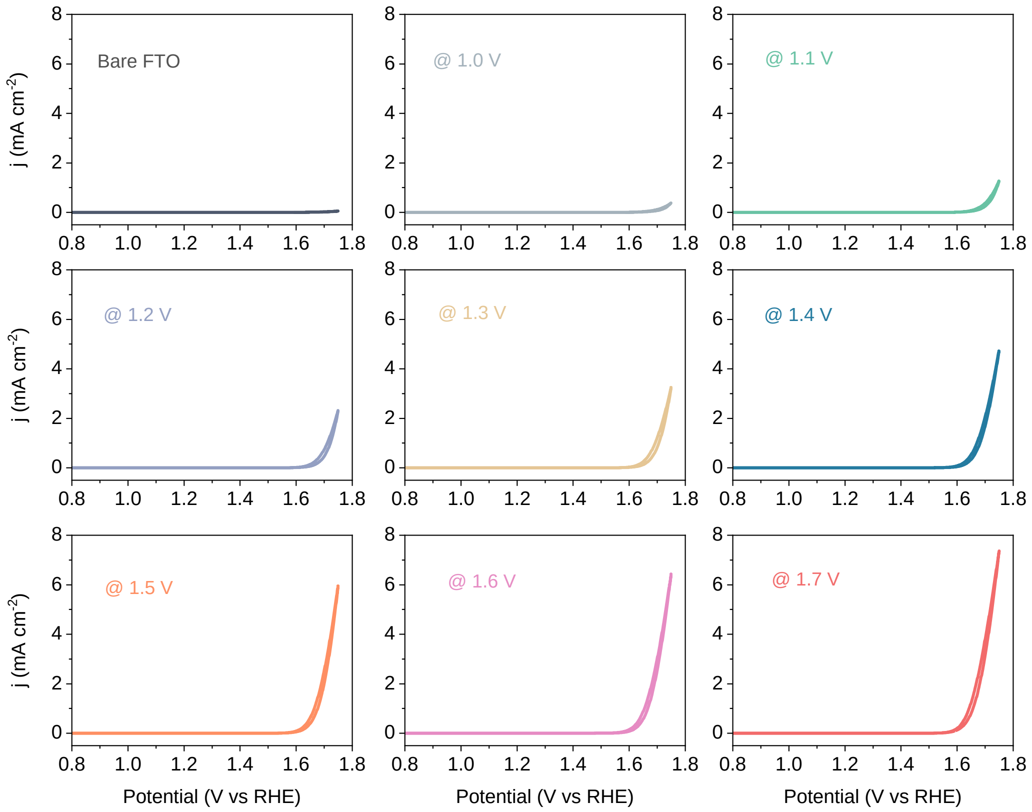

Supplementary Fig. 32 | The CV curves of Co-PH catalysts prepared by 10 min deposition at different potentials. Co-PH catalysts were deposited in $Co(phen)_2(OH)_2$-containing 1.0 M NaOH, and after the deposition, the CV curves of Co-PH catalysts were recorded in phen-free 1.0 M NaOH. Note that the bare FTO has no electrochemical response in the current potential range.

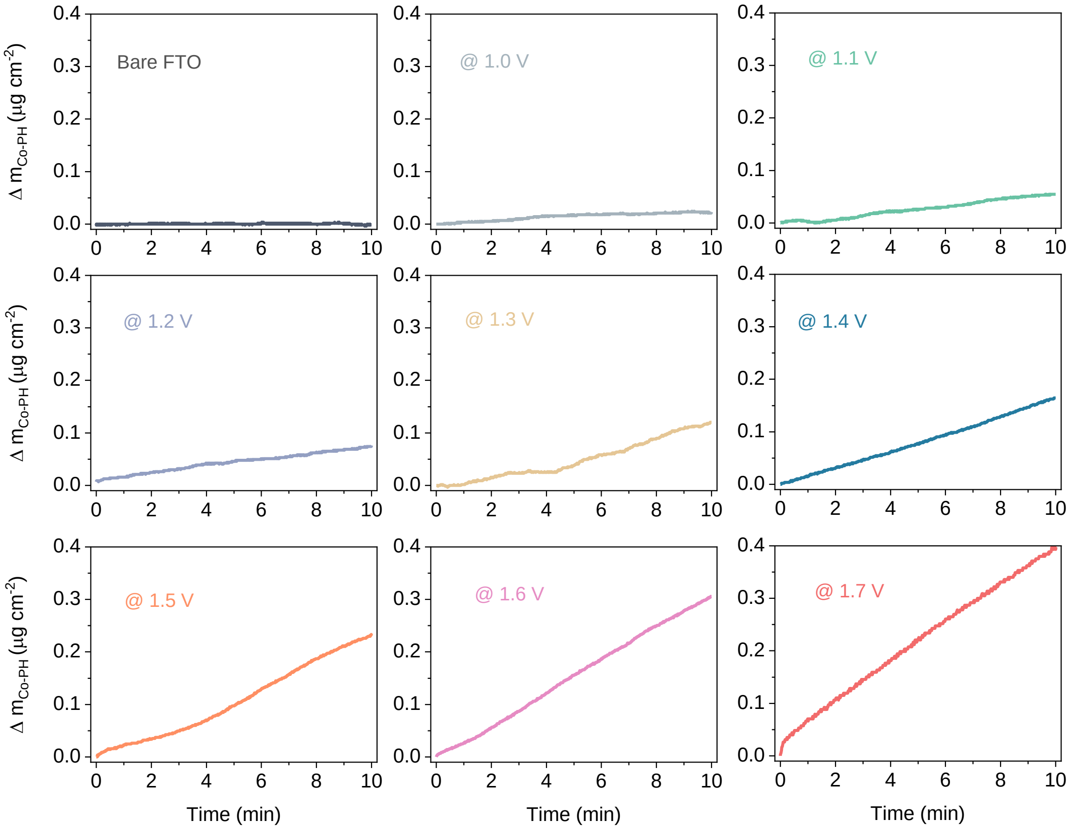

Supplementary Fig. 33 | The mass evolution with time during the Co-PH deposition at different potentials. It is monitored by electrochemical quartz crystal microbalance (EQCM). The Co-PH catalysts were deposited for 10 min in $Co(phen)_2(OH)_2$-containing 1.0 M NaOH from 1.0 V to 1.7 V_{RHE}, respectively. Note that no detectable mass changes are observed under OCP.

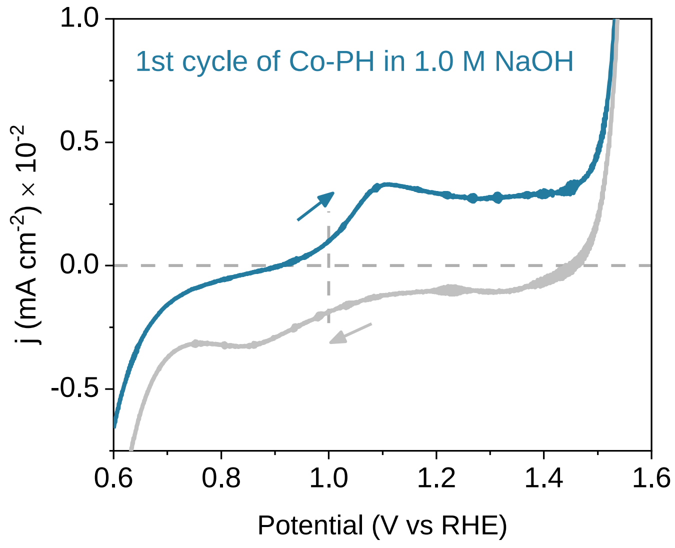

Supplementary Fig. 34 | The first CV of Co-PH in 1.0 M NaOH. The Co-PH was prepared by 10 min deposition on FTO at 1.7 V_{RHE} in $Co(phen)_2(OH)_2$-containing 1.0 M NaOH. The blue curve shows the oxidation process of Co^{2+} to Co^{3+}/Co^{4+}, and the grey one shows the reduction process of Co^{3+}/Co^{4+} to Co^{2+}.

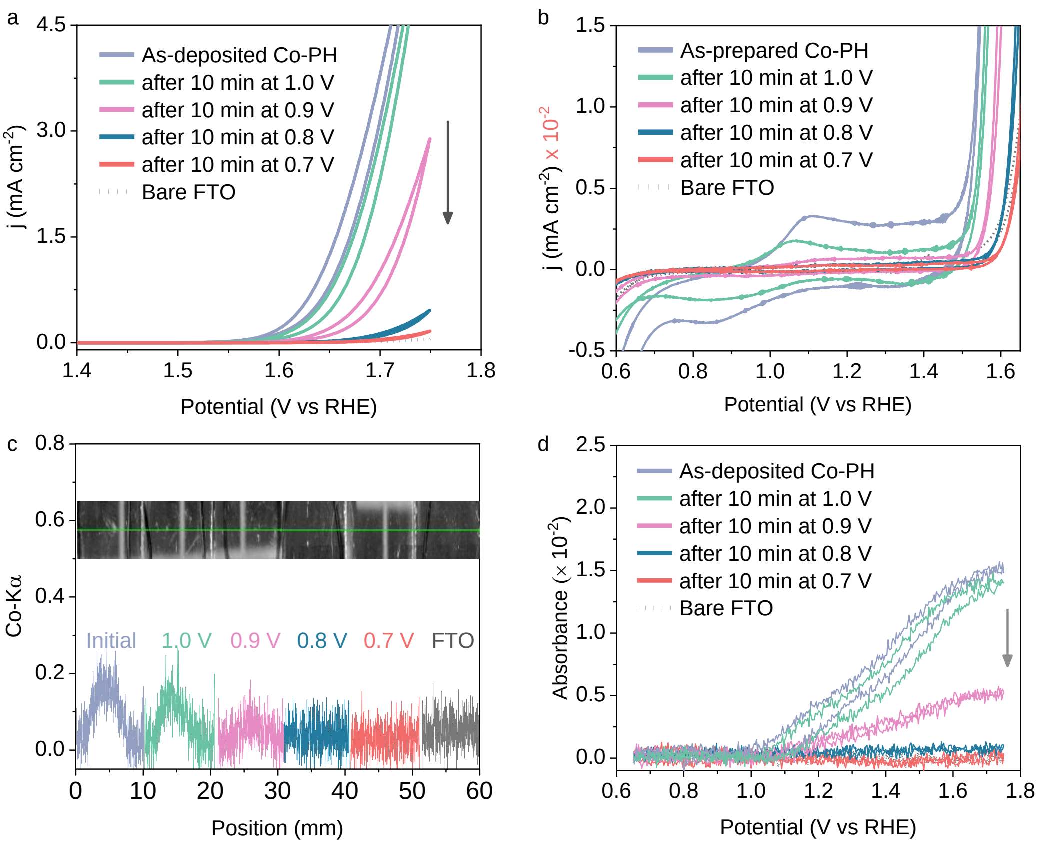

Supplementary Fig. 35 | The valence-dependent dissolution of Co-PH. (a) The CVs of Co-PH in 1.0 M NaOH after 10 min of dissolution at different potentials in $Co(phen)_2(OH)_2$-containing 1.0 M NaOH. **(b)** The corresponding redox couples. **(c)** The linear mapping of Co distribution of Co-PH electrodes by monitoring the Co-K_α emission with XRF. **(d)** The in-situ UV-Vis spectra of Co-PH in 1.0 M NaOH after 10 min reduction in $Co(phen)_2(OH)_2$-containing 1.0 M NaOH at different potentials.

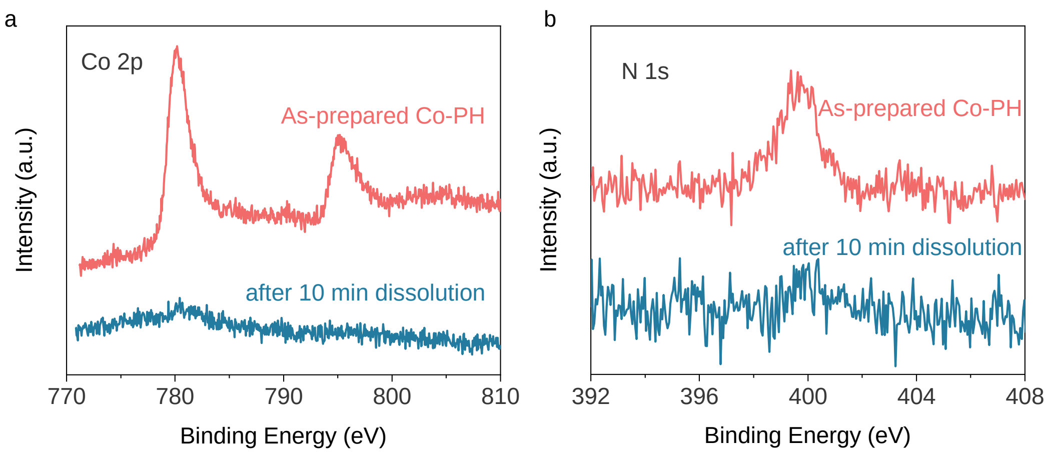

Supplementary Fig. 36 | Monitoring the valence-dependent deposition/dissolution process of Co-PH by XPS. (a) The Co 2*p*, and **(b)** N 1*s* XPS of as-prepared Co-PH by 10 min deposition at 1.7 V_{RHE} and after subsequent 10 min reduction at 0.7 V_{RHE} in $Co(phen)_2(OH)_2$-containing 1.0 M NaOH.

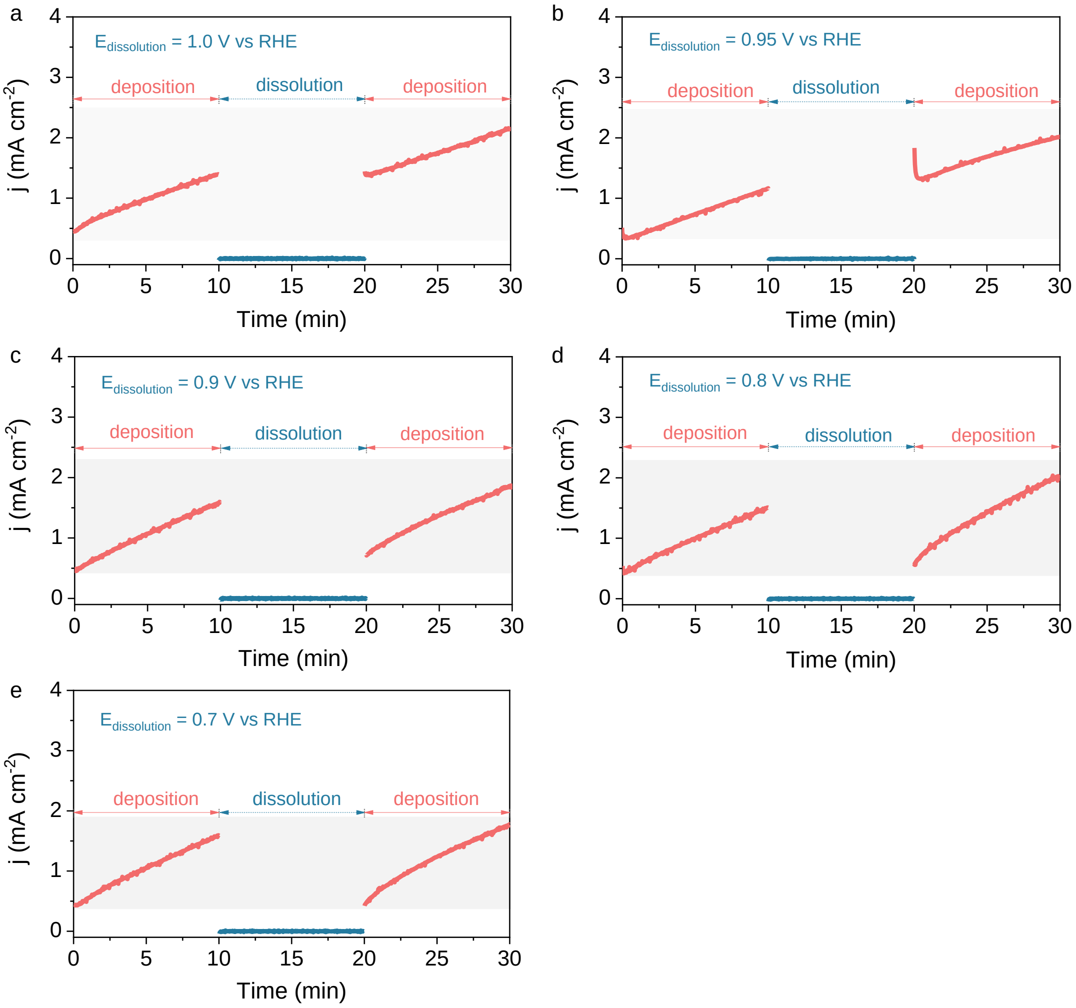

Supplementary Fig. 37 | The valence-dependent deposition/dissolution process of Co-PH. The applied deposition potential (red curves) is 1.7 V_{RHE} and the reduction potentials (blue curves) were setted at **(a)** 1.0 V_{RHE}, **(b)** 0.95 V_{RHE}, **(c)** 0.9 V_{RHE}, **(d)** 0.8 V_{RHE} and **(e)** 0.7 V_{RHE}, respectively. The deposition/dissolution process was implemented in $Co(phen)_2(OH)_2$-containing 1.0 M NaOH.

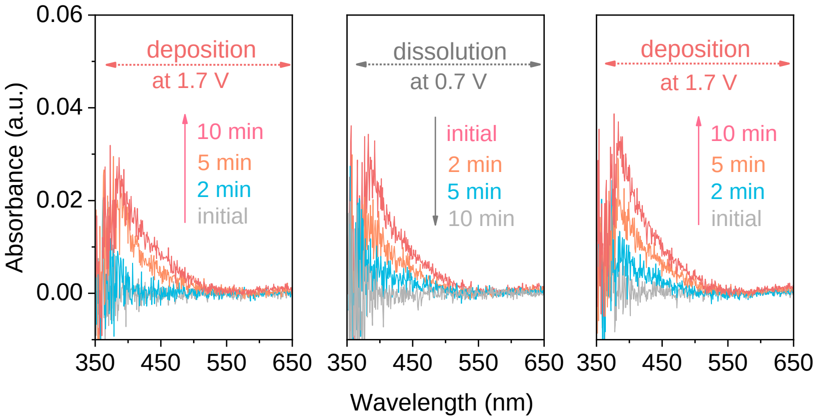

Supplementary Fig. 38 | In-situ UV-Vis tracks the deposition/dissolution process of Co-PH catalysts. The deposition/dissolution process was tracked in $Co(phen)_2(OH)_2$-containing 1.0 M NaOH.

Supplementary discussion to Supplementary Figs. 31-38

Valence-dependent interactions between Co and phen

(1) Valence-dependent deposition. In-situ deposition of phen-containing CoO_xH_y (Co-PH) with abundant Co^{4+} in 1.0 M NaOH containing $Co(phen)_2(OH)_2$ complex during the OER was carried out. Different deposition potentials were applied from 1.0 (that is, close to the onset potential of $Co^{2+/3+}$ in Supplementary Fig. 31) to 1.7 V_{RHE} for 10 min. From the CV curves in Supplementary Fig. 32, we can find a potential-regulated activity relationship. In addition, the mass loadings of Co-PH catalysts in-situ measured by EQCM evidenced a valence-dependent deposition process (Supplementary Fig. 33). Hence, the deposition of Co-PH will take place under applied potential once the Co^{2+} is oxidized to Co^{3+} that can be further easily oxidized to Co^{4+} with the aid of phen.

(2) Valence-dependent coordination and dissolution. A stepwise decrease in reduction potential from 1.0 to 0.7 V_{RHE} (considering the reduction peak of $Co^{3+/2+}$ centered approximately at 0.9 V_{RHE} in Supplementary Fig. 34) leads to different degrees of reduction to Co^{2+}. As expected, attenuation trends in the redox couple are observed. Complete dissolution of Co-PH is observed at 0.7 V_{RHE} (Supplementary Figs. 35-36). Co Kα via X-Ray fluorescence (XRF) line scan and in-situ UV-Vis support the conclusion. Interestingly, multiple deposition-dissolution cycles at an alternating potential between 1.7 and 0.7 V_{RHE} in 1.0 M NaOH electrolyte containing $Co(phen)_2(OH)_2$ demonstrate a quite reversible/regenerable manner (Supplementary Figs. 37-38).

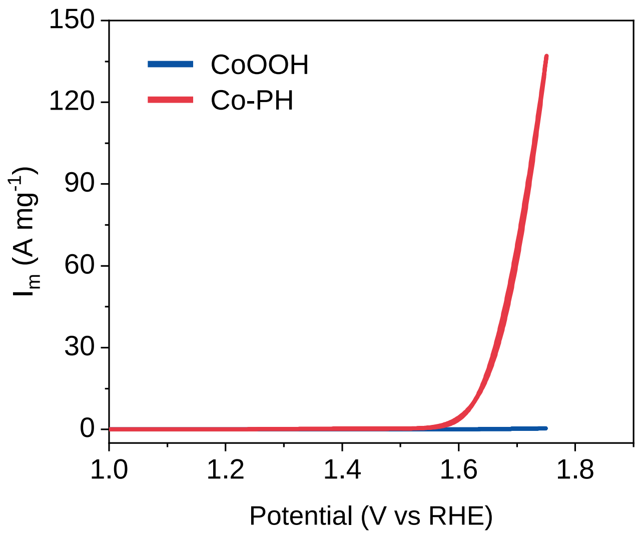

Supplementary Fig. 39 | The mass activity of CoOOH and Co-PH before *iR* correction. Co-PH was freshly deposited for 10 min at 1.7 V_{RHE} in $Co(phen)_2(OH)_2$-containing 1.0 M NaOH. The mass of Co was determined by ICP-MS.

Supplementary Table 6 | The OER TOFs of reported Co-based layered double hydroxides and (oxy) hydroxides at an overpotential of 0.35 V.

Catalyst	TOF_{Co} (s^{-1})	Ref
CoFe LDH	~0.00505	14
CoCo LDH	~0.00265	14
NiCo LDH	~0.00281	14
CoMn LDH	~0.00110	14
$Co(OH)_2$	~0.00052	14
CoOOH	0.007±0.001	15

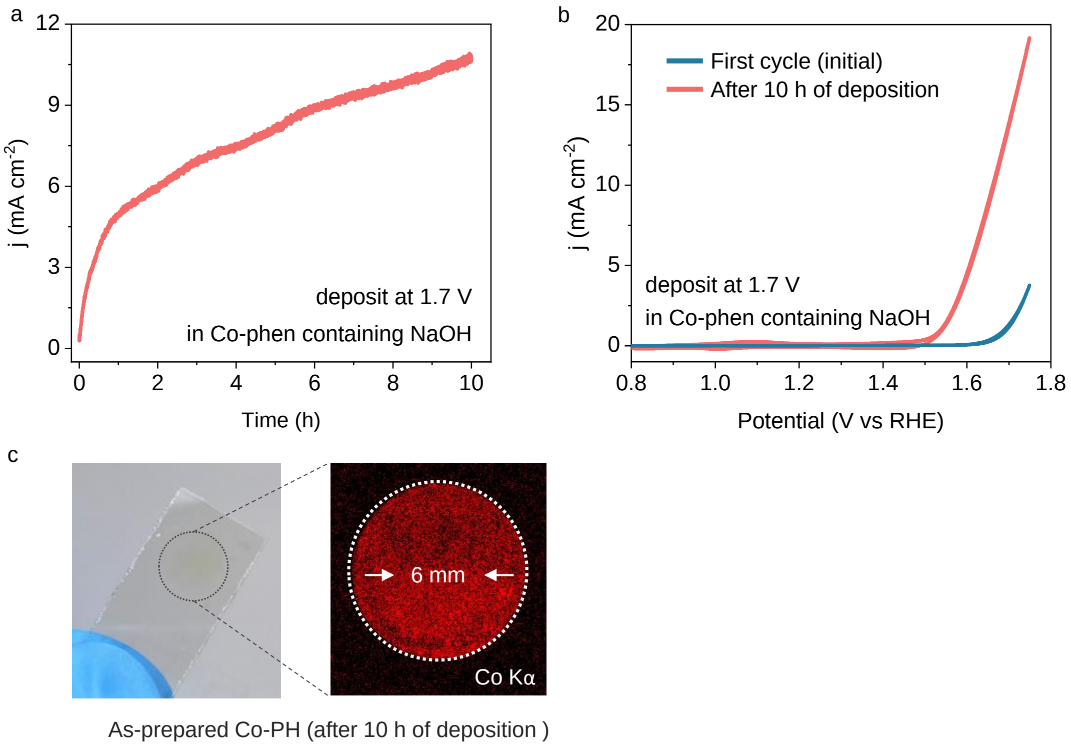

Supplementary Fig. 40 | The deposition of Co-PH. (a) The chronoamperometry curves during the in-situ deposition process of Co-PH on FTO at 1.7 V_{RHE} for 10 h in $Co(phen)_2(OH)_2$-containing 1.0 M NaOH. **(b)** The corresponding CV curves (the first cycle already shows some activity due to the fast deposition of Co-PH). **(c)** The corresponding photographs and XRF mapped Co distribution.

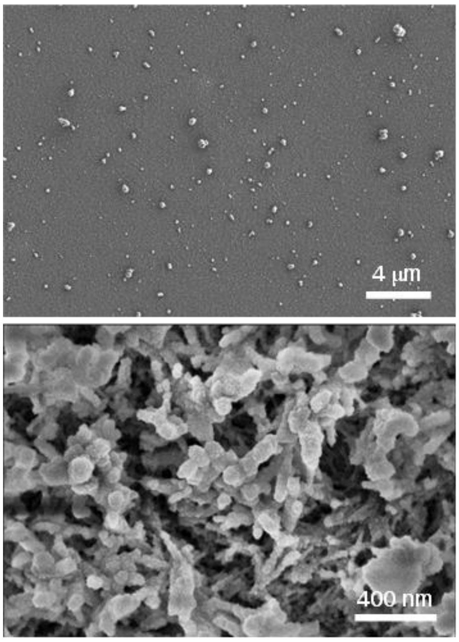

Supplementary Fig. 41 | The surface morphology of Co-PH after 10 h deposition. Co-PH was deposited on FTO at 1.7 V_{RHE} in $Co(phen)_2(OH)_2$-containing 1.0 M NaOH for 10 h.

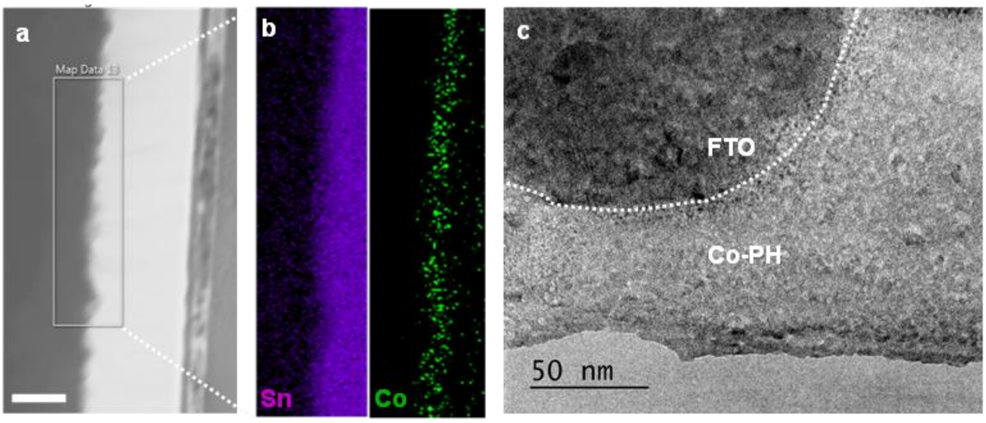

Supplementary Fig. 42 | The cross-section STEM images of Co-PH after 10 h deposition. (a) The cross-section image of Co-PH on the FTO scale bar: 200 nm. **(b)** The corresponding element mapping. **(c)** The cross-section image. The Co-PH was deposited at 1.7 V_{RHE} in $Co(phen)_2(OH)_2$-containing 1.0 M NaOH for 10 h.

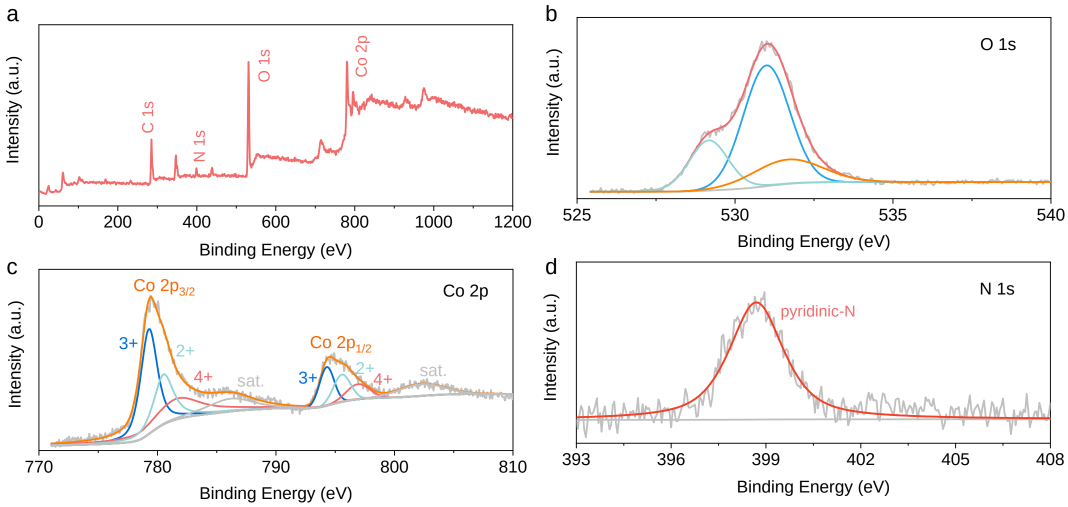

Supplementary Fig. 43 | The XPS of Co-PH after 10 h deposition. (a) The XPS survey, **(b)** O 1s, **(c)** Co 2*p*, and **(d)** N 1*s* spectra of Co-PH after 10 h deposition at 1.7 V_{RHE} in $Co(phen)_2(OH)_2$-containing 1.0 M NaOH. The fitted peaks at 529.2 eV and 531.0 eV are assigned to OH^- and O^{2-}, respectively. The peak at 531.7 eV is associated with Co-O-H[16]. The fitted peaks at 780.5 and 795.6 eV[17-19] are attributed to the Co^{4+}. The peaks at 779.3 and 794.3 eV, and peaks at 781.7 and 796.9 eV are attributed to the Co^{3+} and Co^{2+}, respectively[20]. The fitted peak at 398.6 eV is generally deemed as a pyridinic-N bond[21].

Supplementary Table 7 | The composition of Co-PH after 10 min and 10 h deposition at 1.7 V_{RHE} in $Co(phen)_2(OH)_2$-containing 1.0 M NaOH.

.

	**Co 2*p***	**N 1*s***	**Co/N**
10 min	66.2%	33.8%	1.96
10 h	67.5%	32.5%	2.08

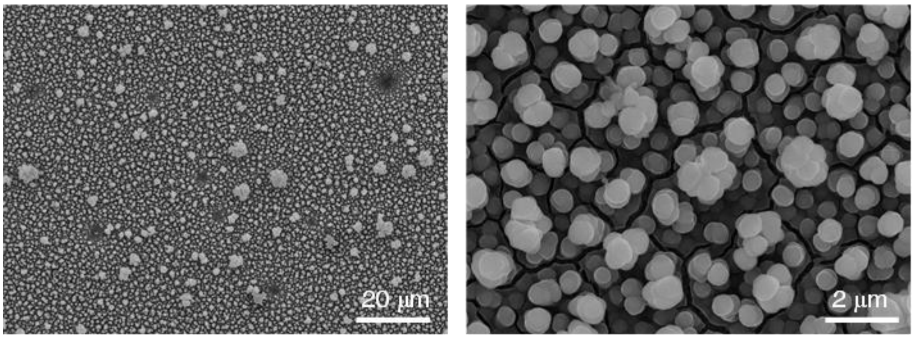

Supplementary Fig. 44 | The surface morphology of Co-PH after 40 h deposition. The Co-PH was deposited on FTO in Co(phen)$_2$(OH)$_2$-containing 1.0 M NaOH at 1.7 V_{RHE} for 40 h.

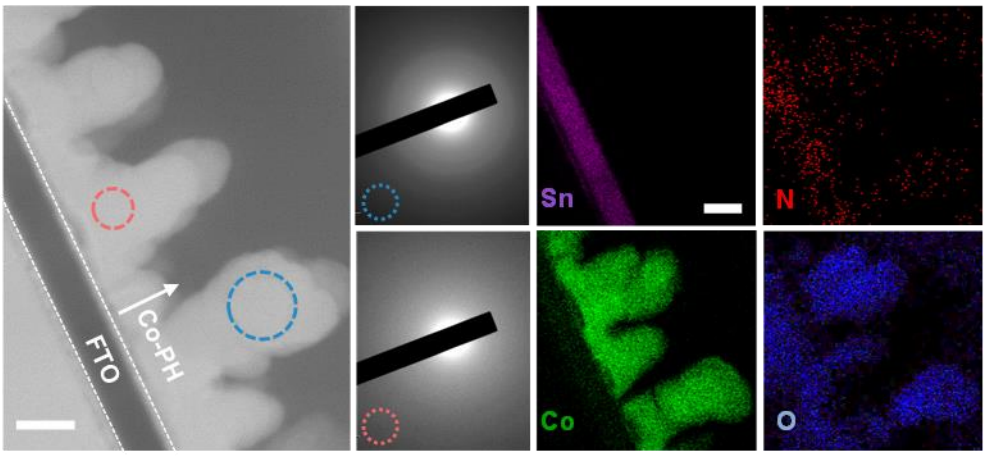

Supplementary Fig. 45 | The cross-section STEM images of Co-PH on FTO after 40 h deposition. The cross-section images, SAED, and corresponding cross-section element mapping of Co-PH film. The Co-PH was deposited in Co(phen)$_2$(OH)$_2$-containing 1.0 M NaOH at 1.7 V_{RHE} for 40 h. Scale bar: 500 nm.

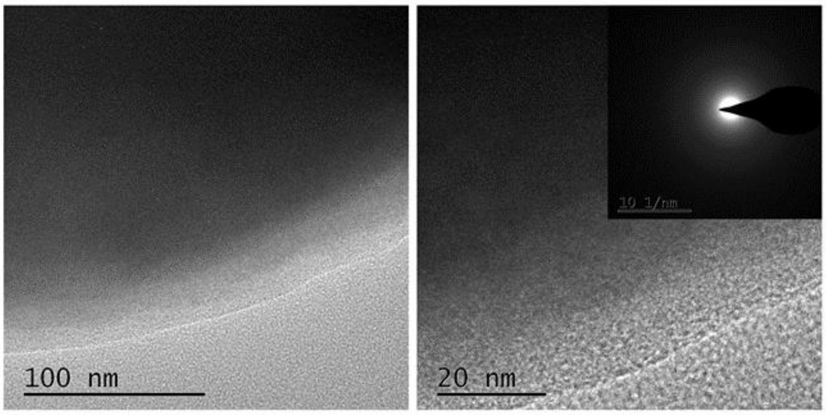

Supplementary Fig. 46 | HRTEM. The HRTEM and electron diffraction (inset) of the Co-PH after 500 h deposition in $Co(phen)_2(OH)_2$-containing 1.0 M NaOH at 10 mA cm^{-2}.

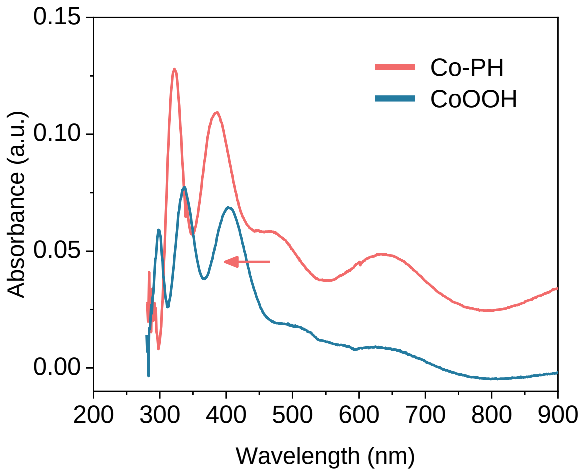

Supplementary Fig. 47 | The UV-Vis spectra. The UV-Vis spectra of CoOOH and Co-PH films on FTO. The absorbance was subtracted by bare FTO. The clear blue shift of absorption bands of Co-PH relative to CoOOH suggests the variation of Co valence states and coordination environments. The Co-PH was deposited in $Co(phen)_2(OH)_2$-containing 1.0 M NaOH at 1.7 V_{RHE} for 10 h.

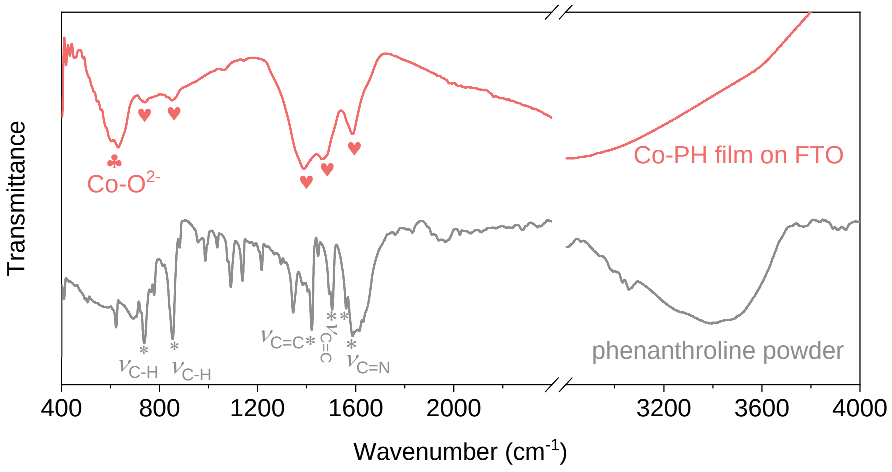

Supplementary Fig. 48 | The ATR-FTIR spectra of Co-PH. The ATR-FTIR of phenanthroline and Co-PH film on FTO. The Co-PH was deposited in $Co(phen)_2(OH)_2$-containing 1.0 M NaOH at 1.7 V_{RHE} for 10 h. The bands at 737 cm^{-1} and 852 cm^{-1} are ascribed to the out-of-plane bending of C-H on benzene and heterocyclic rings, respectively. The peaks at 1421 cm^{-1} and 1504 cm^{-1} are associated with the symmetric and asymmetric stretching of the carbocyclic ring, and the strong bands around 1558 cm^{-1} and 1587 cm^{-1} are assigned to the C=N stretching vibrations[22,23]. These bands are present in Co-PH film with slight shifts, confirming the embedding of phen into Co-PH. Besides, the peak at 632 cm^{-1} is ascribed to $Co\text{-}O^{2-}$ according to previous report[24,25].

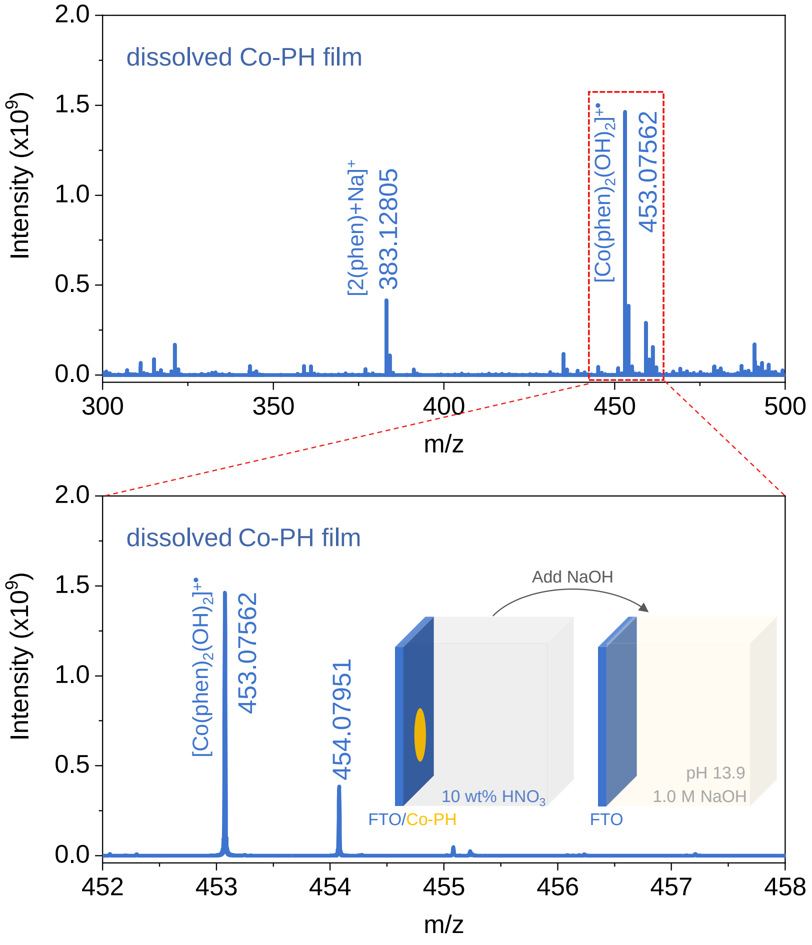

Supplementary Fig. 49 | The FT-ICR-MS spectra of dissolved Co-PH film. The Co-PH film was deposited at 1.7 V_{RHE} in $Co(phen)_2(OH)_2$-containing 1.0 M NaOH for 10 h. For the measurements of FT-ICR-MS, deposited Co-PH film was first soaked in the 10 wt% HNO_3 solutions over 10 h to dissolve. Excess NaOH solid was then added to this solution to adjust the solution pH to 13.9. The obtained solution was used for the FT-ICR-MS characterization. The spectra were recorded using dual electrospray ionization (ESI) source in the positive ion mode.

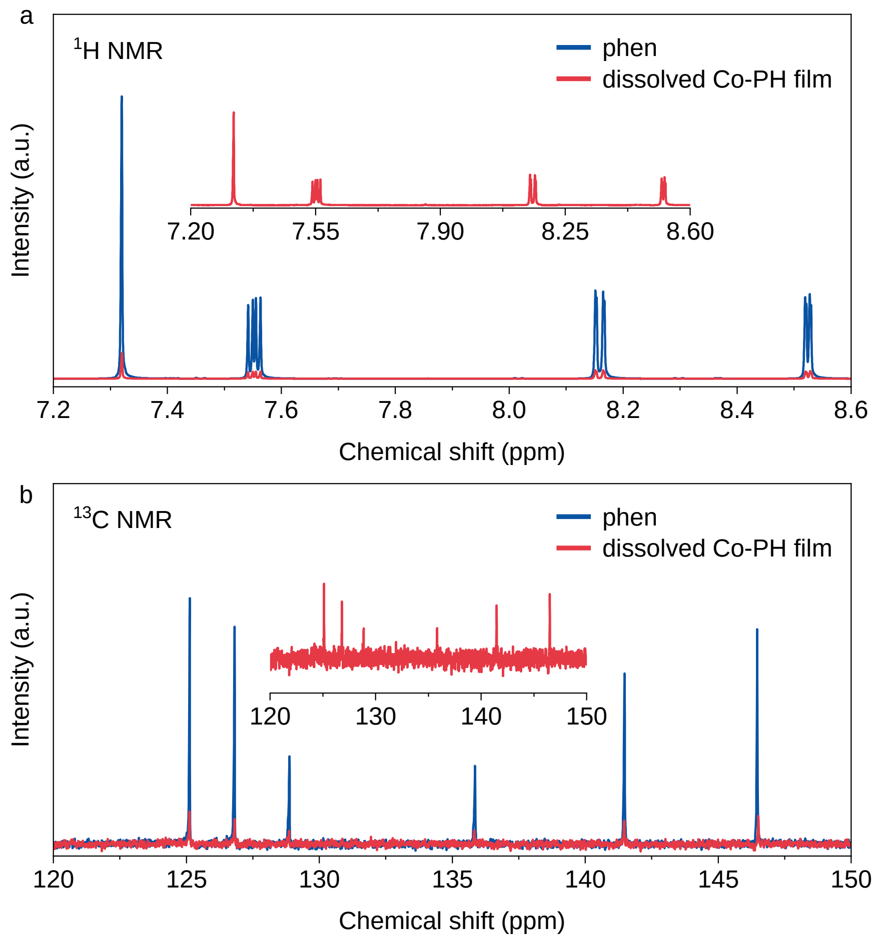

Supplementary Fig. 50 | The NMR spectra of dissolved Co-PH film. (a) The ^{1}H and **(b)** ^{13}C NMR spectra of dissolved Co-PH film. The Co-PH film was deposited at 1.7 V_{RHE} in $Co(phen)_2(OH)_2$-containing 1.0 M NaOH for 10 h. For the measurements of NMR measurements, typically, deposited Co-PH film was first soaked in the 5 wt% HNO_3 solutions containing 95 wt% D_2O over 10 h to dissolve. The obtained solution was used for the NMR measurements. Phen powder was also dissolved in the 5 wt% HNO_3 solutions containing 95 wt% D_2O for NMR tests.

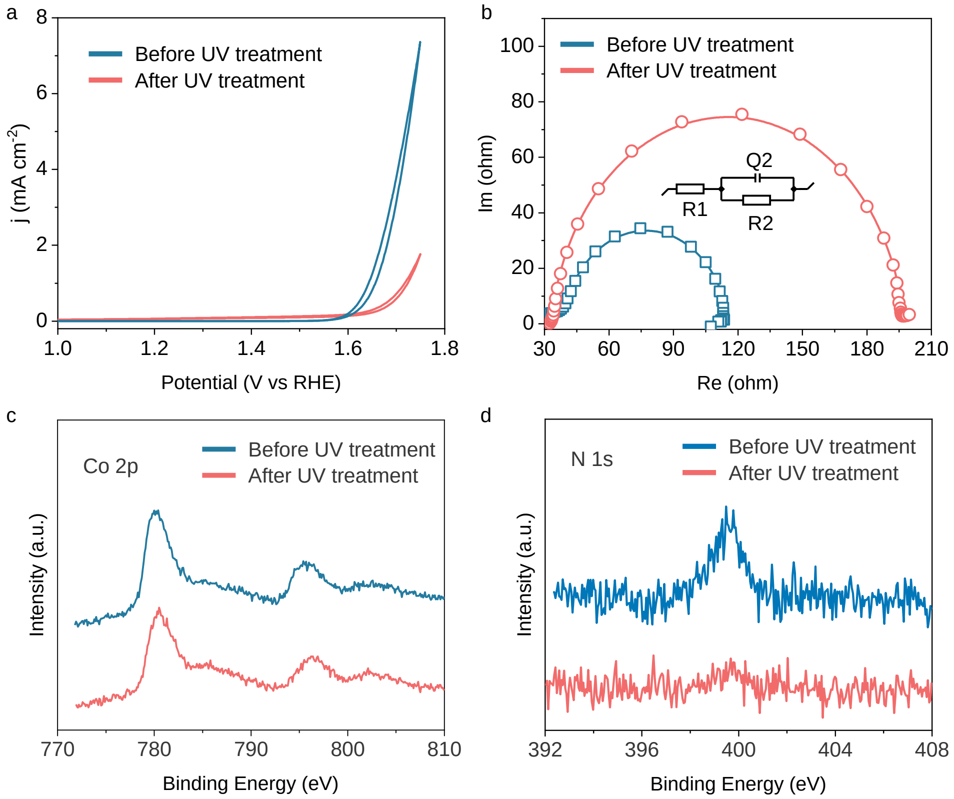

Supplementary Fig. 51 | Decomposition of phen ligand in Co-PH film by UV irradiation. (a) The CVs of Co-PH (prepared by 10 min deposition in Co(phen)$_2$(OH)$_2$-containing 1.0 M NaOH at 1.7 V_{RHE}) in 1.0 M NaOH before and after 20 h of UV irradiation. **(b)** The impedance of Co-PH before and after 20 h of UV irradiation. The inset shows the equivalent circuit containing constant phase elements (Q2) and resistance (R1) through FTO/catalyst and (R2) through catalyst/redox couple. **(c)** The corresponding Co 2*p*, and **(d)** N 1*s* XPS spectra.

Supplementary Table 8 | The R1 and R2 of Co-PH before and after UV irradiation.

	**R1/ohm**	**R2/ohm**
Before UV treatment	38.43	77.04
After UV treatment	33.43	163.5

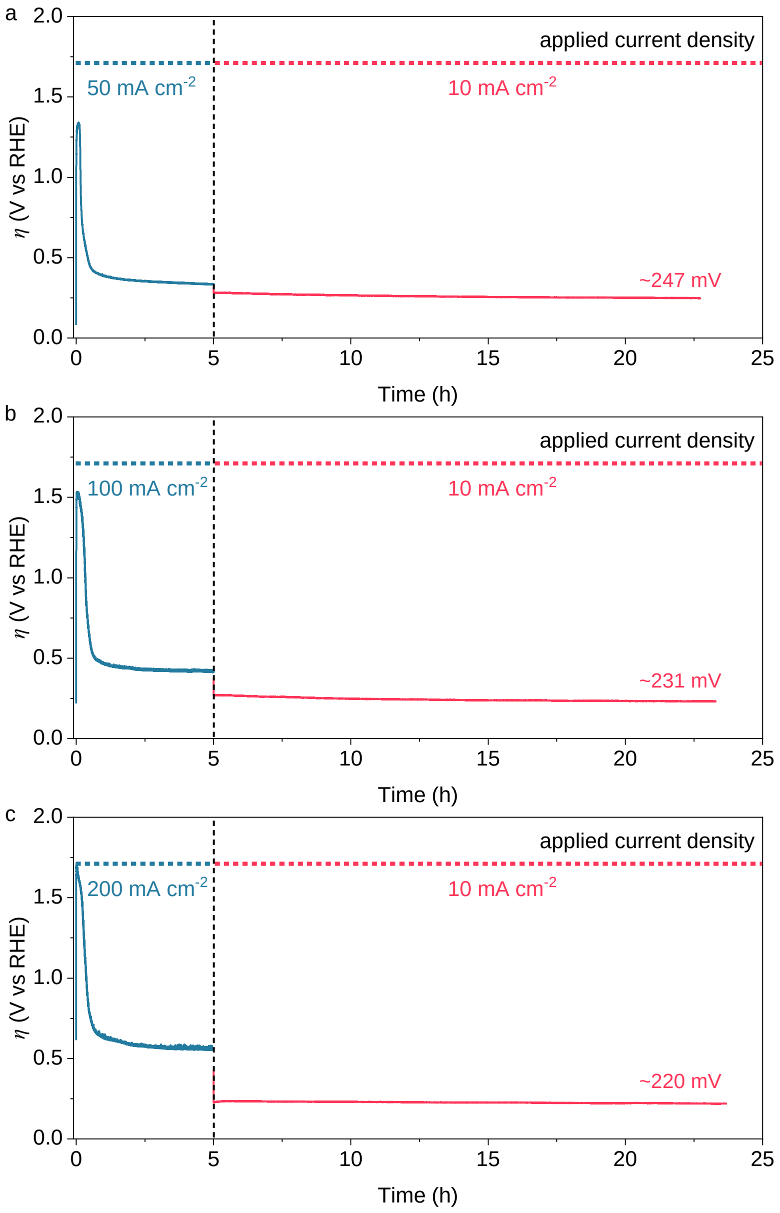

Supplementary Fig. 52 | The stability tests of Co-PH on FTO. The Co-PH was in-situ deposited on bare FTO at **(a)** 50 mA cm^{-2}, **(b)** 100 mA cm^{-2}, **(c)** 200 mA cm^{-2} for 5 h and thereafter operated at 10 mA cm^{-2} in $Co(phen)_2(OH)_2$-containing 1.0 M NaOH, respectively.

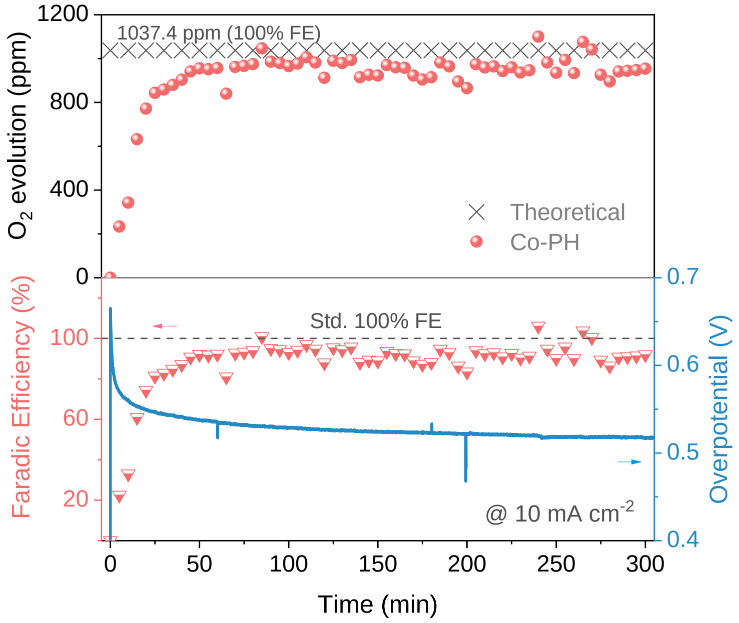

Supplementary Fig. 53 | Online GC monitoring oxygen evolution and faradaic efficiency. The amount of O_2 evolved on Co-PH (upper), and the faradic efficiency along with the OER curve at 10 mA cm^{-2} (lower). The online GC was carried out in a gas-tight H-cell with a typical three-electrode system, and the content of O_2 during the in-situ deposition process of Co-PH on FTO was obtained in Co(phen)$_2$(OH)$_2$-containing 1.0 M NaOH. The generated O_2 was measured at 5 min intervals.

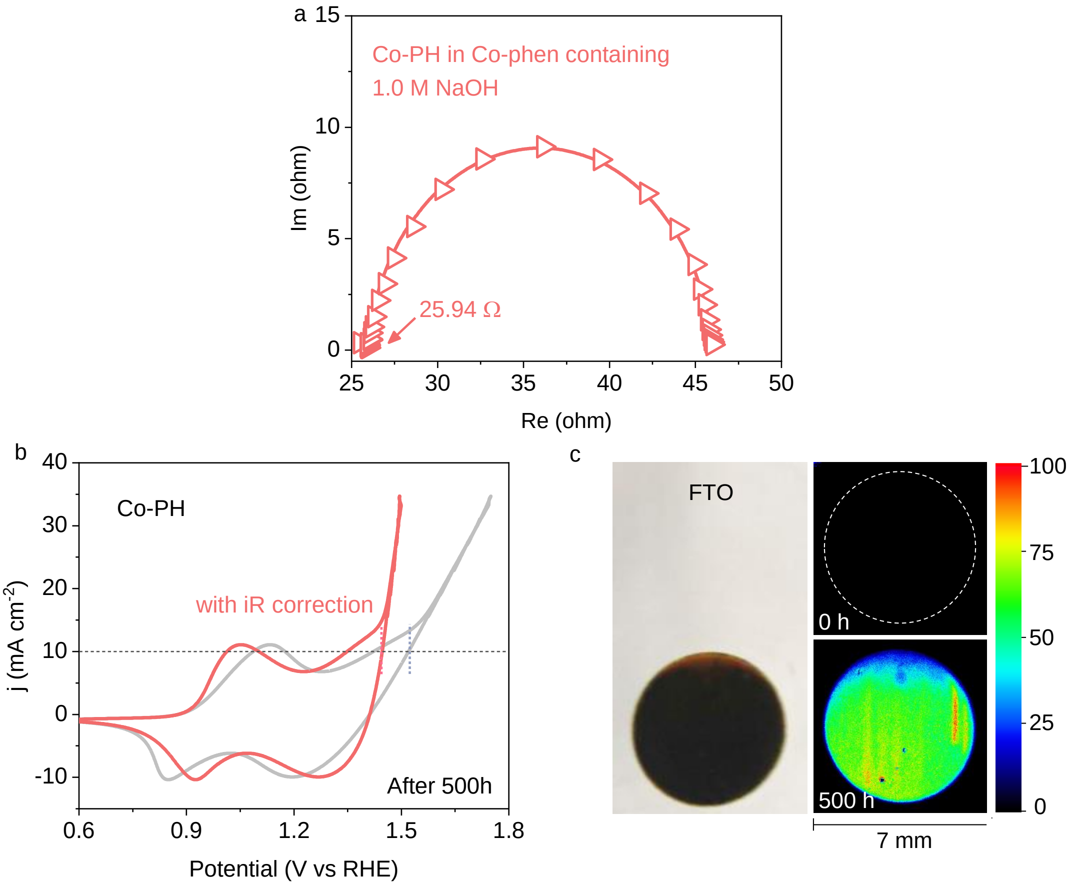

Supplementary Fig. 54 | The effect of 500 h durability test at 10 mA cm^{-2}. (a) The ohmic resistance of Co-PH on FTO after 500 h of deposition/operation in $Co(phen)_2(OH)_2$-containing 1.0 M NaOH. **(b)** The corresponding CV curves. Noting that the overpotential at 10 mA cm^{-2} was acquired from the backward scan. **(c)** The corresponding photographs and composition mapping (by XRF) of Co-PH after the 500 h stability test. The color bar indicates the distribution of the Co element.

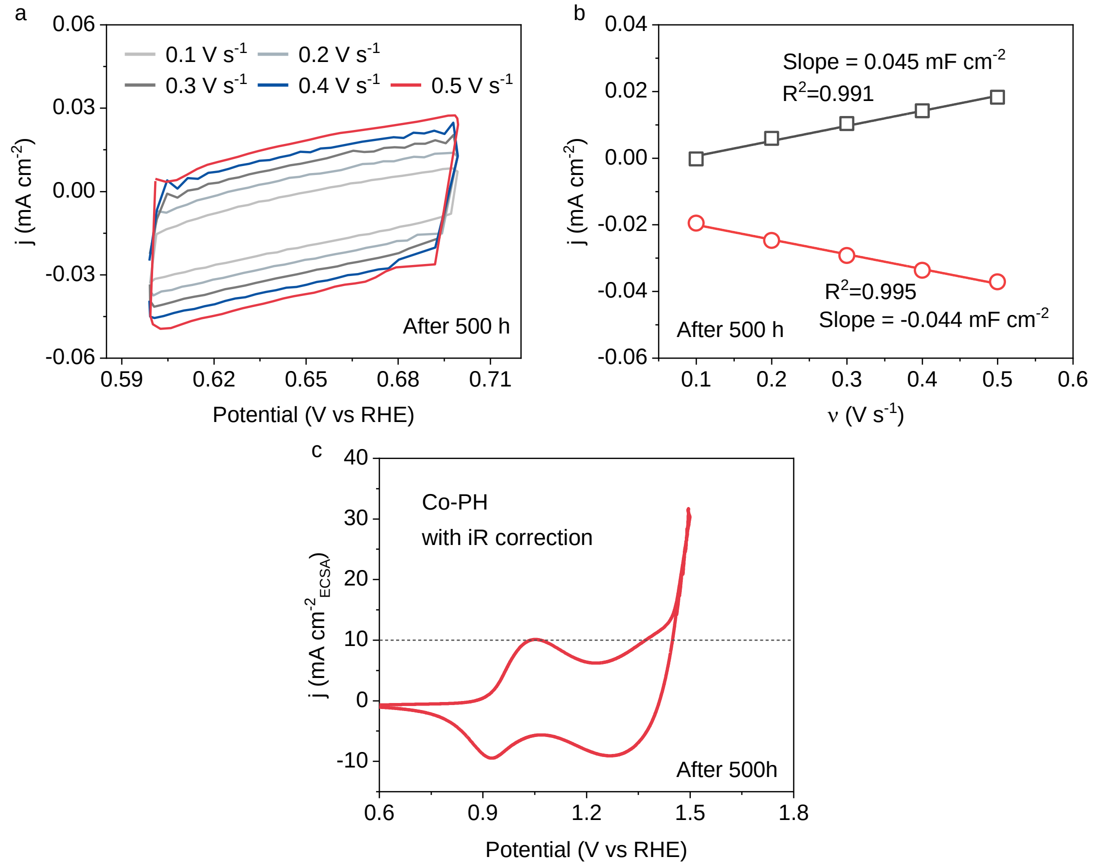

Supplementary Fig. 55 | The intrinsic activity of Co-PH on FTO with *iR* correction after 500 h of operation at 10 mA cm^{-2}. (a) CV curves of Co-PH (after 500 h of deposition in a non-Faradic region (0.6 ~ 0.7 V)) in 1.0 M NaOH at a scan rate of 0.1 V s^{-1} to 0.5 V s^{-1}. **(b)** Liner fitting of the cathodic and anodic charging current density at 0.65 V_{RHE} as a function of scan rate. **(c)** The ECSA-normalized CV curves of Co-PH after 500 h of deposition. Noting that the overpotential at 10 mA cm^{-2} was acquired from the backward scan.

Supplementary Table 9 | The comparison of activity and stability of Co-based catalysts in alkaline electrolytes.

Catalyst	Electrolyte	Substrate	η_{geo}	η_{ECSA}	Stability	Ref.
Co-PH	1 M NaOH	FTO	216	218	> 1600 h	This work
Co-PH	1 M NaOH	FTO	237 @ 20 mA cm^{-2}	239 @ 20 mA cm^{-2}	NA	This work
Co-PH	1 M NaOH	FTO	260 @ 30 mA cm^{-2}	263 @ 30 mA cm^{-2}	NA	This work
CoOOH	0.1 M KOH	Au	550	NA	NA	26
Porous Co-P	1 M KOH	Co foam	NA	380 @ 0.0269 mA cm^{-2}	~3000 h	27
Ag doped CoOOH	1 M KOH	Au	256	NA	~ 2 h	28
Cu doped CoOOH	1 M KOH	Cu	322	NA	NA	28
Fe-Co_3O_4 HHNPs	1 M KOH	GCE	262	300 @ 0.033 mA cm^{-2}	~ 50 h	29
Co-TiO_2	1 M KOH	CP	332	NA	~ 45 h	30
CoCo-NS	1 M KOH	GCE	~360	NA	~ 13 h	31
NiCo NS	1 M KOH	GCE	~325	~330	~ 13 h	31
CoMn LDH	1 M KOH	GCE	324	NA	~ 14 h	32
CoFe LDH	0.1 M KOH	GCE	404	NA	NA	33
CoFe LDH	1 M KOH	GCE	321	NA	NA	34
FeCo LDH	1 M KOH	GCE	334	NA	NA	35
FeCo (0.38% Fe^{2+})	1 M KOH	CP	266	NA	NA	36
Boronized NiFe	1 M KOH	NiFe sheet	~270	309	~3000 h	37
3DGN/CoAl-NS	1 M KOH	GCE	250	NA	~ 30 h	38
$LiCoCl_{1.8}Cl_{0.2}$	1 M KOH	GCE	290 @ 20 mA cm^{-2}	NA	~500 h	39
$LiCoCl_{1.8}Cl_{0.2}$	1 M KOH	GCE	280	280	NA	39
G-FeCoW	1 M KOH	Au	315	NA	NA	35
G-FeCoW	1 M KOH	FTO	314 @ 1.0 mA cm^{-2}	NA	NA	35
G-FeCoW	1 M KOH	GCE	223	NA	NA	35
G-FeCoW	1 M KOH	Au-plated Ni foam	191 @ 30 mA cm^{-2}	NA	~500 h	35
CoFeO@BP	1 M KOH	GCE	266	NA	~ 24 h	40
$CoSe_2$	1 M NaOH	GCE	~380	NA	~ 8 h	41
$CoSe_2$-D_{Fe}-V_{Co}	1 M NaOH	GCE	~310	NA	~ 8 h	41
NiCoP/C	1 M KOH	GCE	~330	NA	~ 10 h	42
$(Ni_2Co_1)_{0.925}Fe_{0.075}$-MOF	1 M KOH	GCE	257	NA	~ 35 h	43

Ni(Fe)OOH-FeS_x	1 M KOH	Ni foam	220	NA	~ 16 h	44
FeCoMoW	1 M KOH	CP	212	NA	NA	36

Note: The overpotential and stability are obtained at 10 mA cm^{-2} if there are no specific notes.

FTO: Fluorine-doped tin oxide glass

CP: Carbon paper.

GCE: Glass carbon electrode.

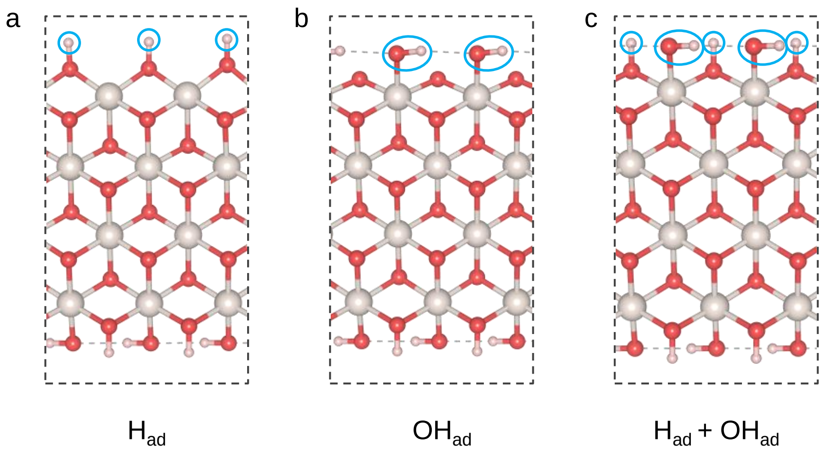

Supplementary Fig. 56 | Structures of different surface phases on CoO_2. (a) surface O sites are adsorbed with H (H_{ad}) to form bridge OH species; **(b)** surface Co sites are adsorbed with atop OH (OH_{ad}), and **(c)** both H_{ad} and OH_{ad} are existing. The adsorbates of surface phases are highlighted by light blue circles on the top views.

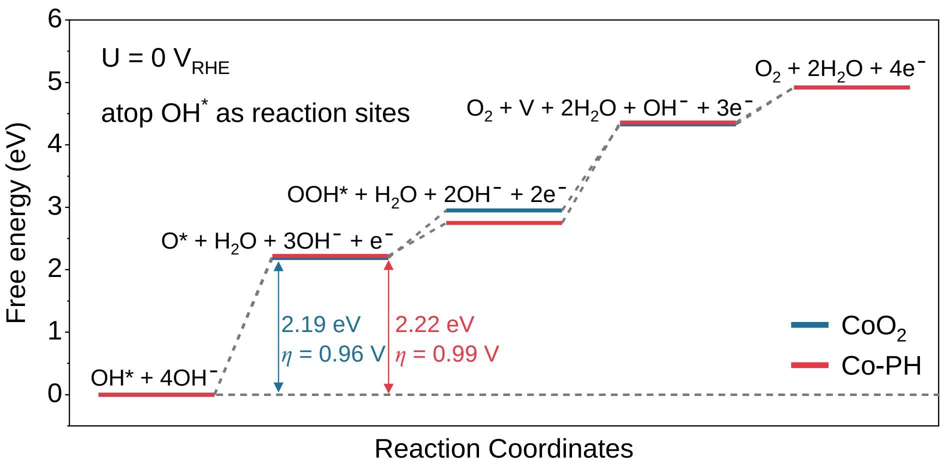

Supplementary Fig. 57 | The reaction free-energy diagrams for OER on CoO_2 and Co-PH with atop OH^* as reaction centers at 0 V_{RHE}.

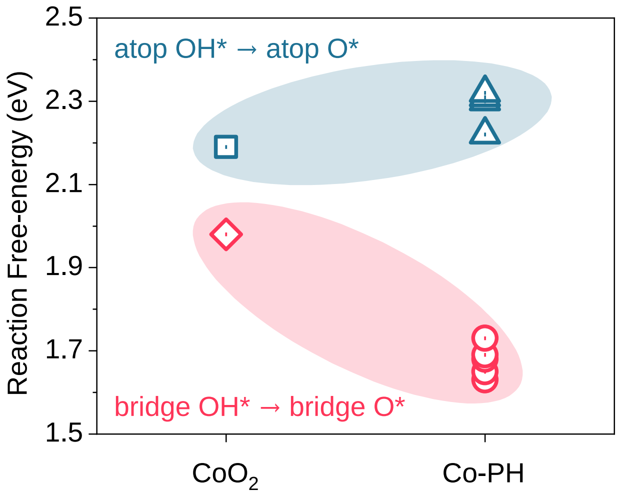

Supplementary Fig. 58 | The reaction free-energy for deprotonation of atop OH^* and bridged OH^* on CoO_2 and Co-PH.

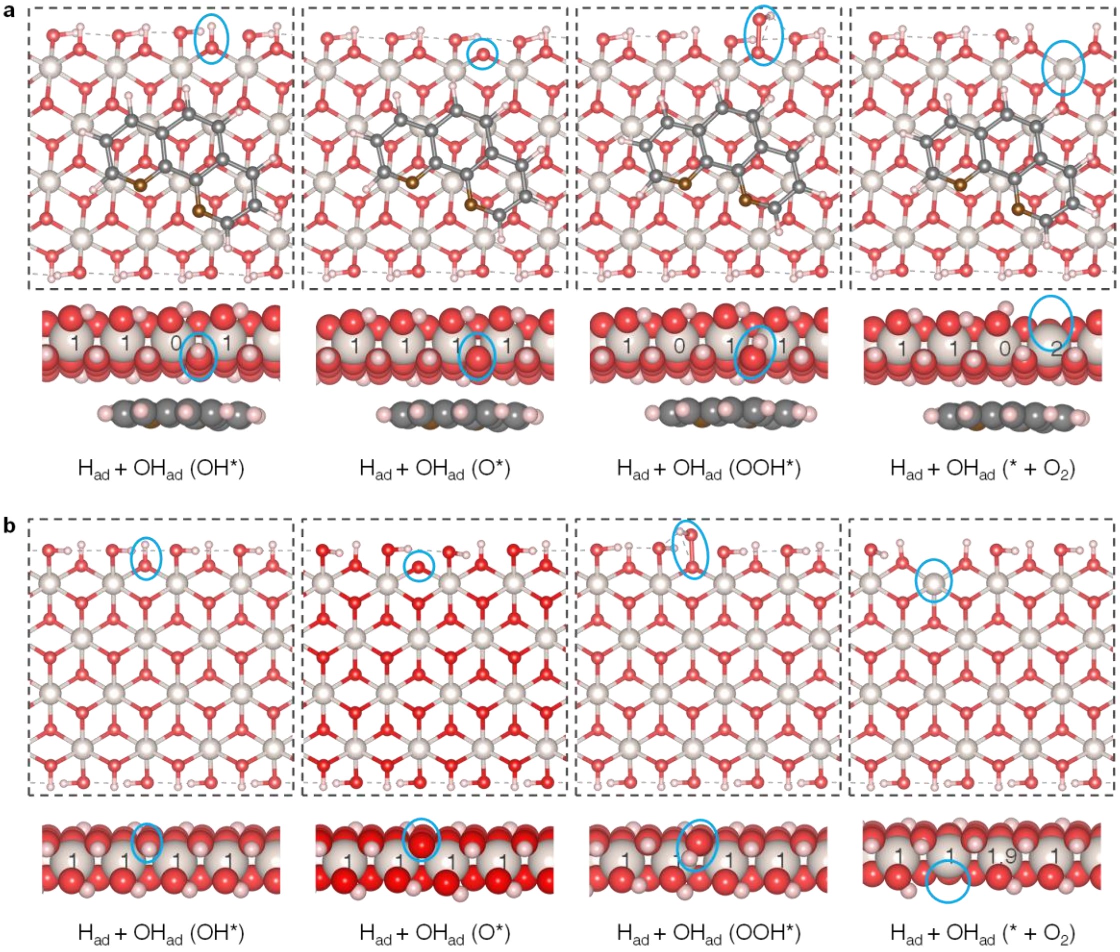

Supplementary Fig. 59 | Structures of reaction intermediates for OER on Co-PH and CoO_2. The structures of reaction intermediates for OER on **(a)** Co-PH and **(b)** CoO_2. The adsorbates are highlighted by light blue circles on the top views (upper) and side views (under). The number labeled on atoms are their magnetic moments in the Bohr magnetic (μ_B). The magnetic moment of Co^{4+} is 1 μ_B, and the magnetic moment of 0 μ_B or 1.9 μ_B indicates the Co^{3+}.

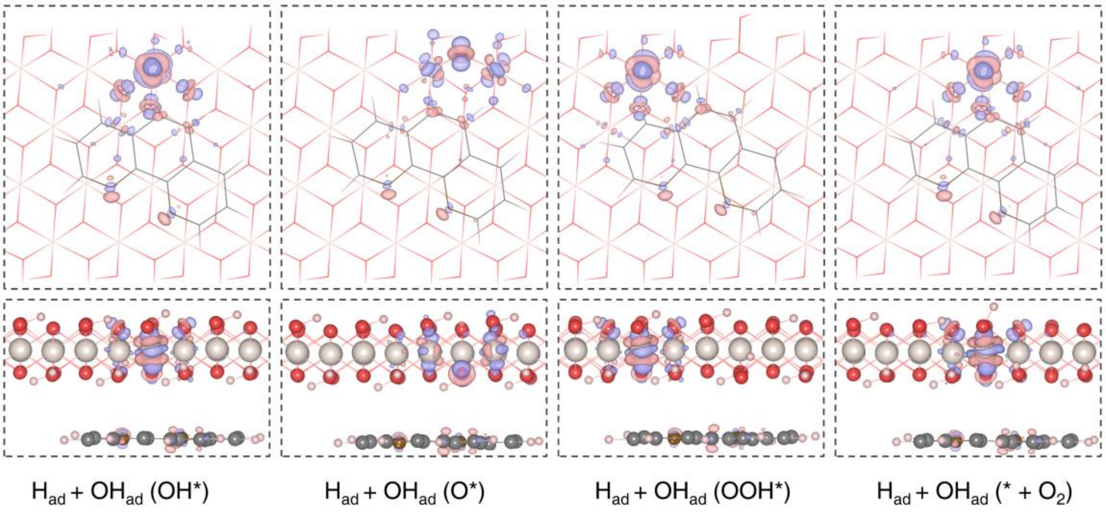

Supplementary Fig. 60 | Charge density differences of Co-PH during the OER process. The upper one is the top view and the bottom one is the side view. The purple isosurfaces correspond to charge densities of 0.011 e/Bohr^3 and represent an increase in the total charge density. The orange isosurfaces correspond to charge densities of -0.011 e/Bohr^3 and represent a decrease in the total charge density.

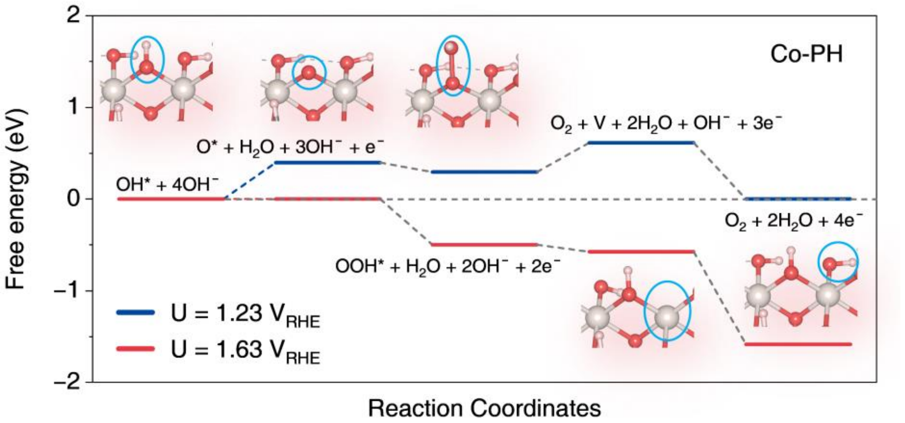

Supplementary Fig. 61 | Reaction free-energy diagrams for OER on Co-PH at different potentials. The reaction free energies at 1.23 V_{RHE} are in blue, and 1.63 V_{RHE} are in red. Insets are the corresponding enlarged models.

Supplementary Table 10 | Magnetic moment (μ_B) of Co atoms on the different reaction sites of the Co-PH OER models which are corresponding to the models in Supplementary Figs. 58-65.

**Co^{1}-O^{5}-Co^{2}**	Co^{1}	Co^{2}	Co^{3}	Co^{4}	Co^{5}	Co^{6}	Co^{7}	Co^{8}	Co^{9}	Co^{10}	Co^{11}	Co^{12}	Co^{13}	Co^{14}	Co^{15}	Co^{16}
**OH***	1	1	1	0	1	1	1	1	1	1	1	1	1	1	1	1
**O***	1	1	1	1	1	1	1	1	1	1	1	1	1	1	1	1
**OOH***	1	1	1	0	1	1	1	1	1	1	1	1	1	1	1	1
**V**	1	2	1	1	1	1	1	1	1	1	1	0	1	1	1	1
**Co^{3}-O^{6}-Co^{1}**	Co^{1}	Co^{2}	Co^{3}	Co^{4}	Co^{5}	Co^{6}	Co^{7}	Co^{8}	Co^{9}	Co^{10}	Co^{11}	Co^{12}	Co^{13}	Co^{14}	Co^{15}	Co^{16}
**OH***	1	1	1	0	1	1	1	1	1	1	1	1	1	1	1	1
**O***	1	1	1	1	1	1	1	1	1	1	1	1	1	1	1	1
**OOH***	1	0	1	1	1	1	1	1	1	1	1	1	1	1	1	1
**V**	2	1	1	0	1	1	1	1	1	1	1	1	1	1	1	1
**Co^{2}-O^{7}-Co^{4}**	Co^{1}	Co^{2}	Co^{3}	Co^{4}	Co^{5}	Co^{6}	Co^{7}	Co^{8}	Co^{9}	Co^{10}	Co^{11}	Co^{12}	Co^{13}	Co^{14}	Co^{15}	Co^{16}
**OH***	1	1	1	0	1	1	1	1	1	1	1	1	1	1	1	1
**O***	1	1	1	1	1	1	1	1	1	1	1	1	1	1	1	1
**OOH***	1	1	0	1	1	1	1	1	1	1	1	1	1	1	1	1
**V**	1	0	1	2	1	1	1	1	1	1	1	1	1	1	1	1
**Co^{3}-O^{8}-Co^{4}**	Co^{1}	Co^{2}	Co^{3}	Co^{4}	Co^{5}	Co^{6}	Co^{7}	Co^{8}	Co^{9}	Co^{10}	Co^{11}	Co^{12}	Co^{13}	Co^{14}	Co^{15}	Co^{16}
**OH***	1	1	1	0	1	1	1	1	1	1	1	1	1	1	1	1
**O***	1	1	1	1	1	1	1	1	1	1	1	1	1	1	1	1
**OOH***	1	0	1	1	1	1	1	1	1	1	1	1	1	1	1	1
**V**	1	1	2	0	1	1	1	1	1	1	1	1	1	1	1	1
**Co^{13}-O^{33}-Co^{14}**	Co^{1}	Co^{2}	Co^{3}	Co^{4}	Co^{5}	Co^{6}	Co^{7}	Co^{8}	Co^{9}	Co^{10}	Co^{11}	Co^{12}	Co^{13}	Co^{14}	Co^{15}	Co^{16}
**OH***	1	1	1	0	1	1	1	1	1	1	1	1	1	1	1	1
**O***	1	1	1	1	1	1	1	1	1	1	1	1	1	1	1	1
**OOH***	0	1	1	1	1	1	1	1	1	1	1	1	1	1	1	1
**V**	1	1	1	1	1	1	1	1	1	1	1	1	1	2	1	0
**Co^{14}-O^{34}-Co^{15}**	Co^{1}	Co^{2}	Co^{3}	Co^{4}	Co^{5}	Co^{6}	Co^{7}	Co^{8}	Co^{9}	Co^{10}	Co^{11}	Co^{12}	Co^{13}	Co^{14}	Co^{15}	Co^{16}
**OH***	1	1	1	0	1	1	1	1	1	1	1	1	1	1	1	1
**O***	1	1	1	1	1	1	1	1	1	1	1	1	1	1	1	1
**OOH***	1	1	1	0	1	1	1	1	1	1	1	1	1	1	1	1
**V**	1	1	1	1	1	1	1	1	1	1	1	1	1	2	1	0
**Co^{15}-O^{35}-Co^{16}**	Co^{1}	Co^{2}	Co^{3}	Co^{4}	Co^{5}	Co^{6}	Co^{7}	Co^{8}	Co^{9}	Co^{10}	Co^{11}	Co^{12}	Co^{13}	Co^{14}	Co^{15}	Co^{16}
**OH***	1	1	1	0	1	1	1	1	1	1	1	1	1	1	1	1
**O***	1	1	1	1	1	1	1	1	1	1	1	1	1	1	1	1
**OOH***	1	1	1	1	1	1	1	1	0	1	1	1	1	1	1	1
**V**	1	1	1	1	1	1	1	1	1	1	1	1	0	1	2	1
**Co^{16}-O^{36}-Co^{13}**	Co^{1}	Co^{2}	Co^{3}	Co^{4}	Co^{5}	Co^{6}	Co^{7}	Co^{8}	Co^{9}	Co^{10}	Co^{11}	Co^{12}	Co^{13}	Co^{14}	Co^{15}	Co^{16}
**OH***	1	1	1	0	1	1	1	1	1	1	1	1	1	1	1	1
**O***	1	1	1	1	1	1	1	1	1	1	1	1	1	1	1	1
**OOH***	1	1	1	0	1	1	1	1	1	1	1	1	1	1	1	1
**V**	1	1	1	1	1	1	1	1	1	1	1	1	0	1	1	2

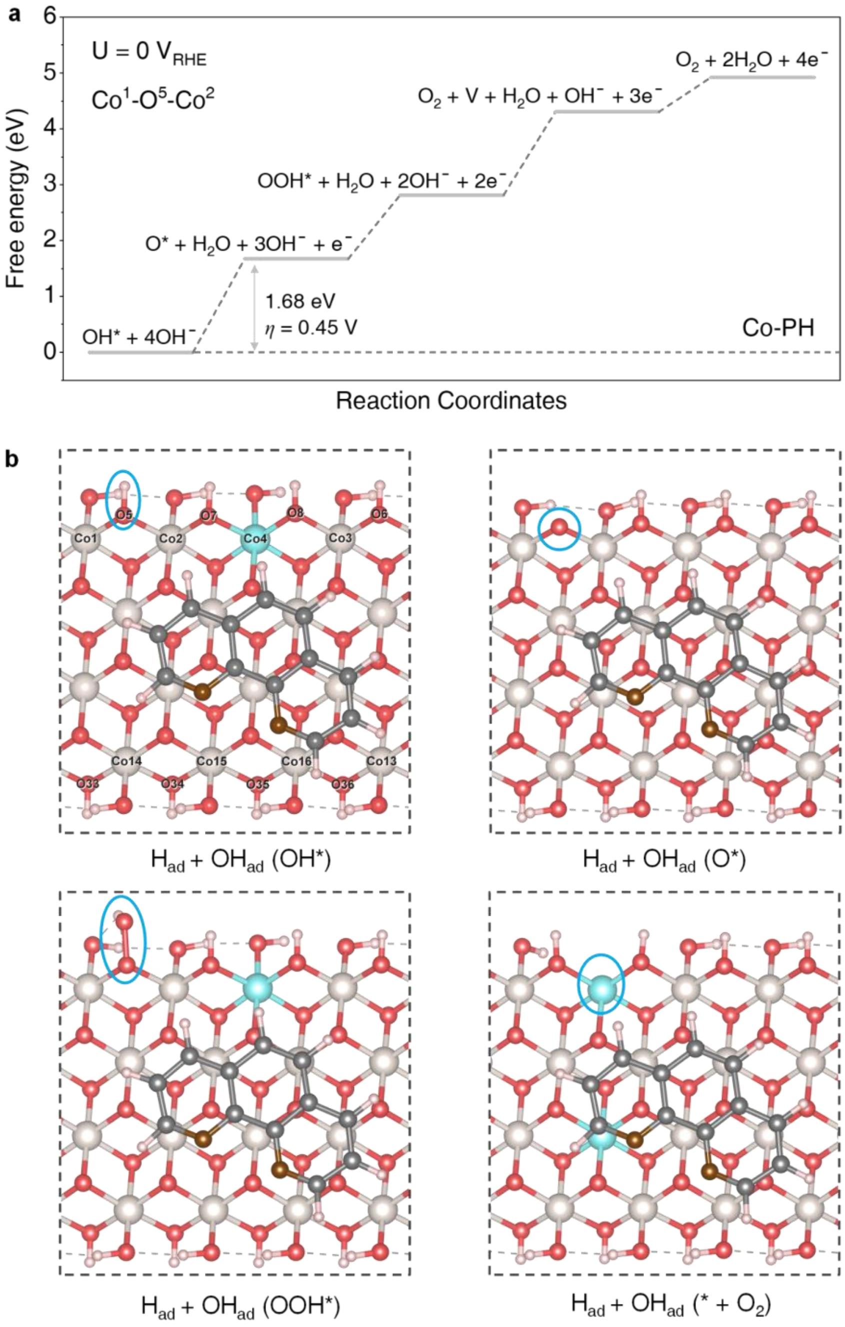

Supplementary Fig. 62 | Reaction free-energy diagram of the Co^1-O^5-Co^2 reaction site on the Co-PH at 0 V_{RHE}. (a) The reaction free-energy diagram on the **Co^1-O^5-Co^2** reaction site on Co-PH and (b) the corresponding models. The reaction sites are shown with light blue circles. Co^{4+}, Co^{3+}, N, C, O, and H are represented by silver, cyan, chocolate, gray, red, and pink balls, respectively.

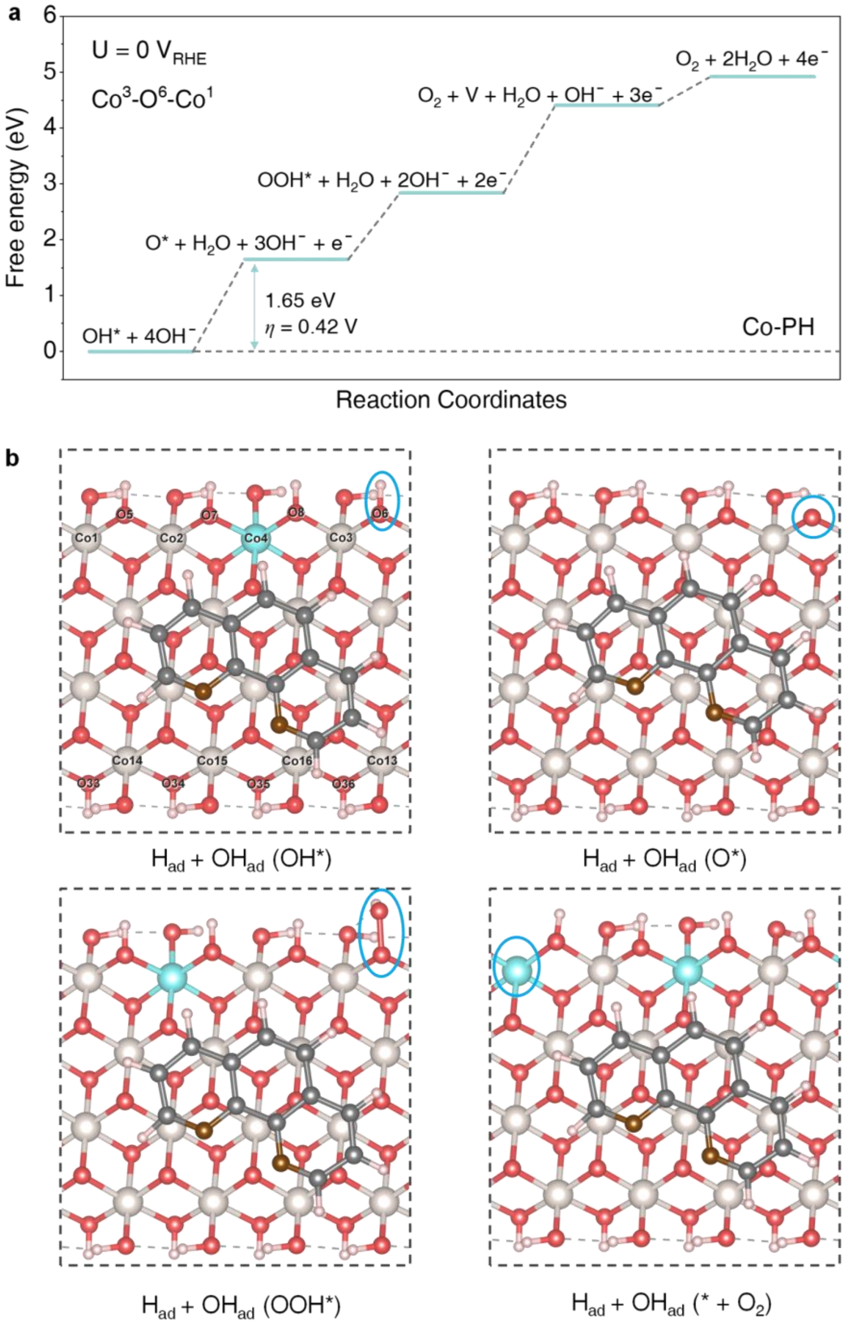

Supplementary Fig. 63 | Reaction free-energy diagram of the Co^3-O^6-Co^1 reaction site on the Co-PH at 0 V_{RHE}. (a) The reaction free-energy diagram on the **Co^3-O^6-Co^1** reaction site on Co-PH and (b) the corresponding models. The reaction sites are shown with light blue circles. Co^{4+}, Co^{3+}, N, C, O, and H are represented by silver, cyan, chocolate, gray, red, and pink balls, respectively.

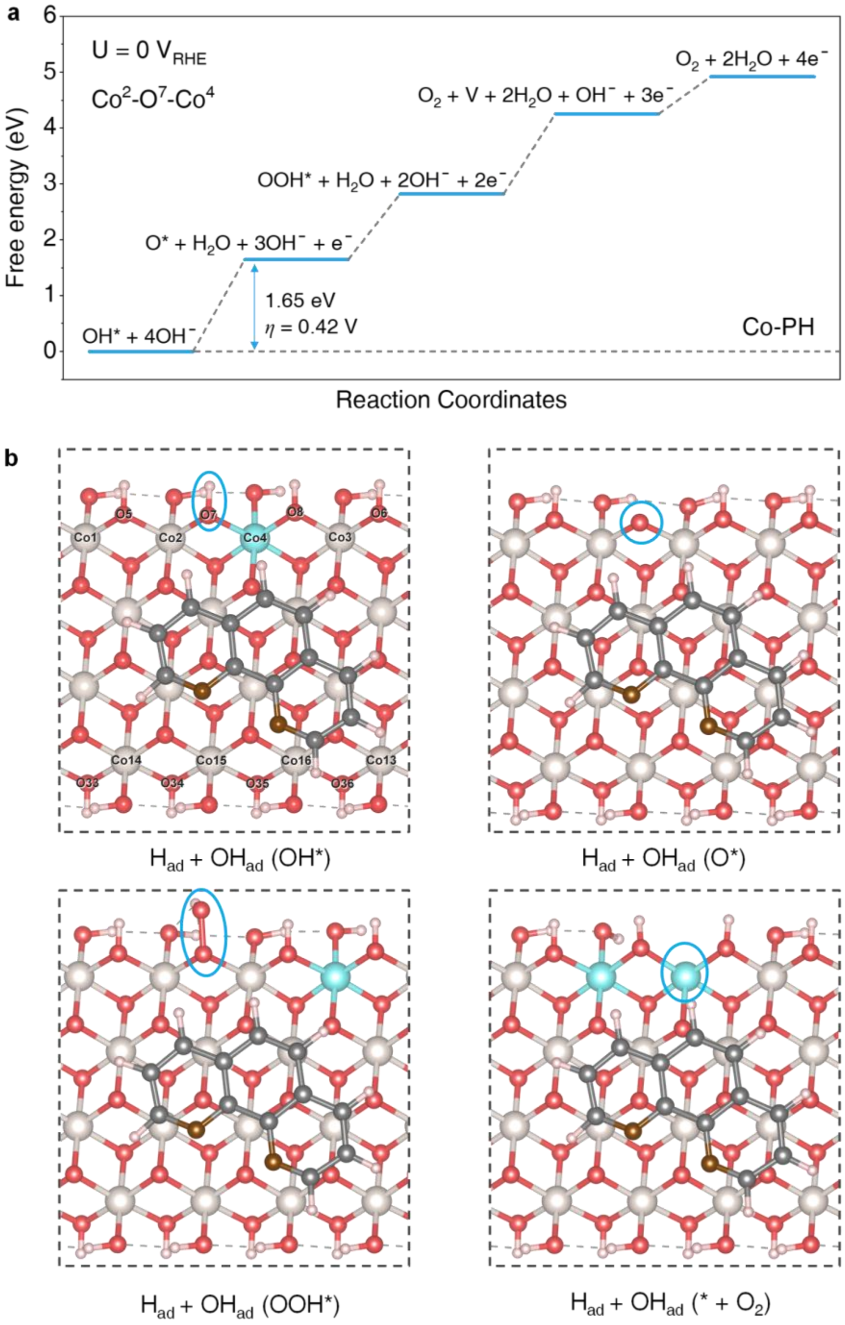

Supplementary Fig. 64 | Reaction free-energy diagram of the Co^2-O^7-Co^4 reaction site on the Co-PH at 0 V_{RHE}. (a) The reaction free-energy diagram on the **Co^2-O^7-Co^4** reaction site on Co-PH and (b) the corresponding models. The reaction sites are shown with light blue circles. Co^{4+}, Co^{3+}, N, C, O, and H are represented by silver, cyan, chocolate, gray, red, and pink balls, respectively.

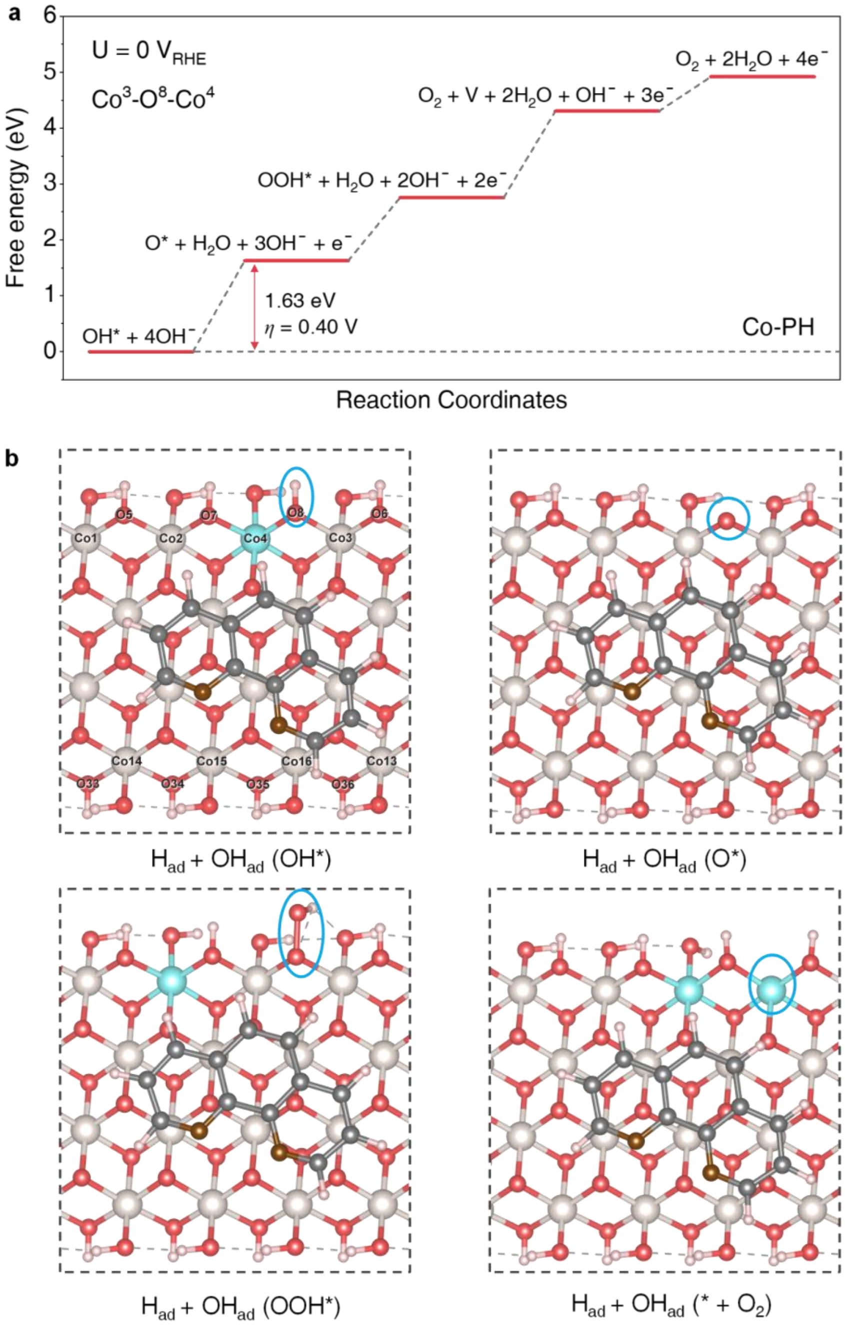

Supplementary Fig. 65 | Reaction free-energy diagram of the Co^3-O^8-Co^4 reaction site on the Co-PH at 0 V_{RHE}. (a) The reaction free-energy diagram on the **Co^3-O^8-Co^4** reaction site on Co-PH and (b) the corresponding models. The reaction sites are shown with light blue circles. Co^{4+}, Co^{3+}, N, C, O, and H are represented by silver, cyan, chocolate, gray, red, and pink balls, respectively.

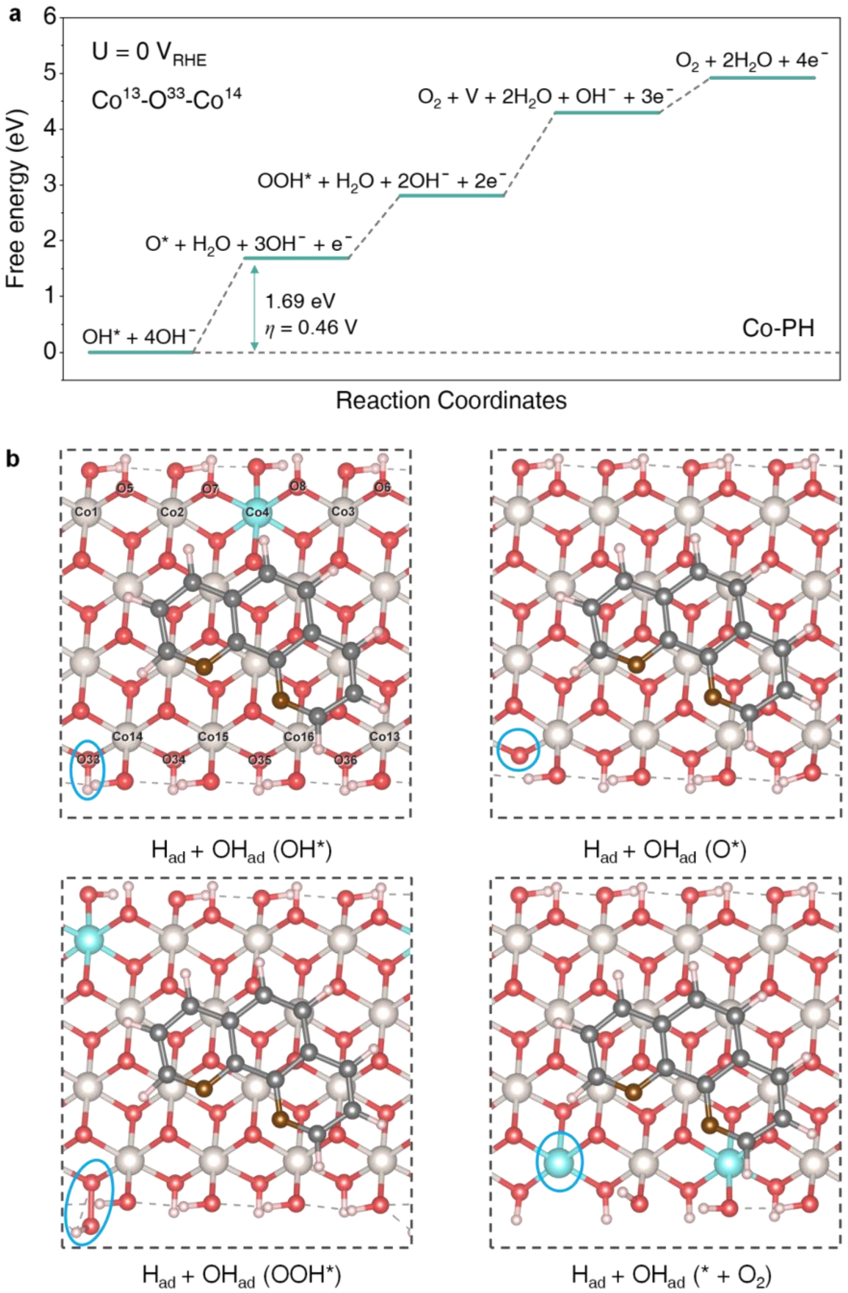

Supplementary Fig. 66 | Reaction free-energy diagram of the Co^{13}-O^{33}-Co^{14} reaction site on the Co-PH at 0 V_{RHE}. (a) The reaction free-energy diagram on the **Co^{13}-O^{33}-Co^{14}** reaction site on Co-PH and (b) the corresponding models. The reaction sites are shown with light blue circles. Co^{4+}, Co^{3+}, N, C, O, and H are represented by silver, cyan, chocolate, gray, red, and pink balls, respectively.

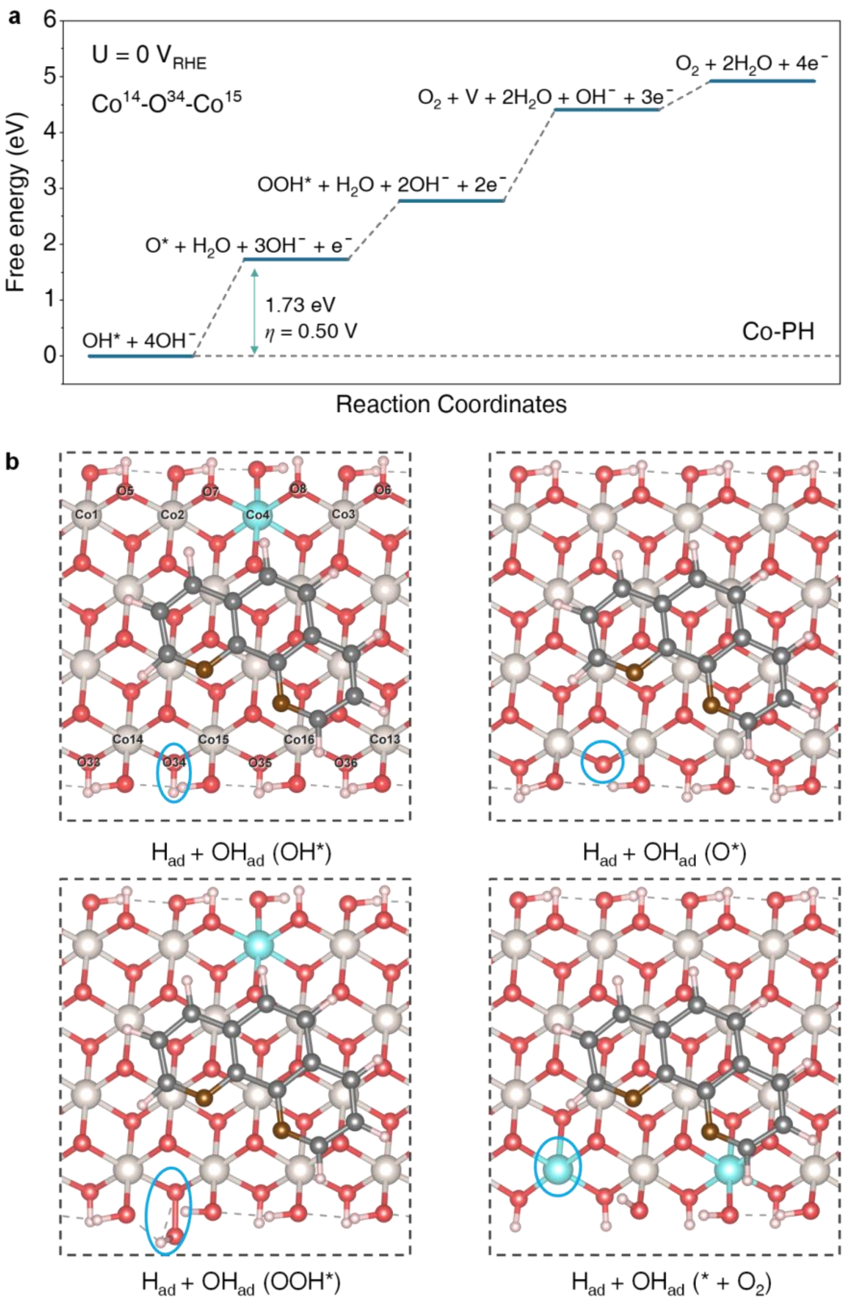

Supplementary Fig. 67 | Reaction free-energy diagram of the Co^{14}-O^{34}-Co^{15} reaction site on the Co-PH at 0 V_{RHE}. (a) The reaction free-energy diagram on the **Co^{14}-O^{34}-Co^{15}** reaction site on Co-PH and (b) the corresponding models. The reaction sites are shown with light blue circles. Co^{4+}, Co^{3+}, N, C, O, and H are represented by silver, cyan, chocolate, gray, red, and pink balls, respectively.

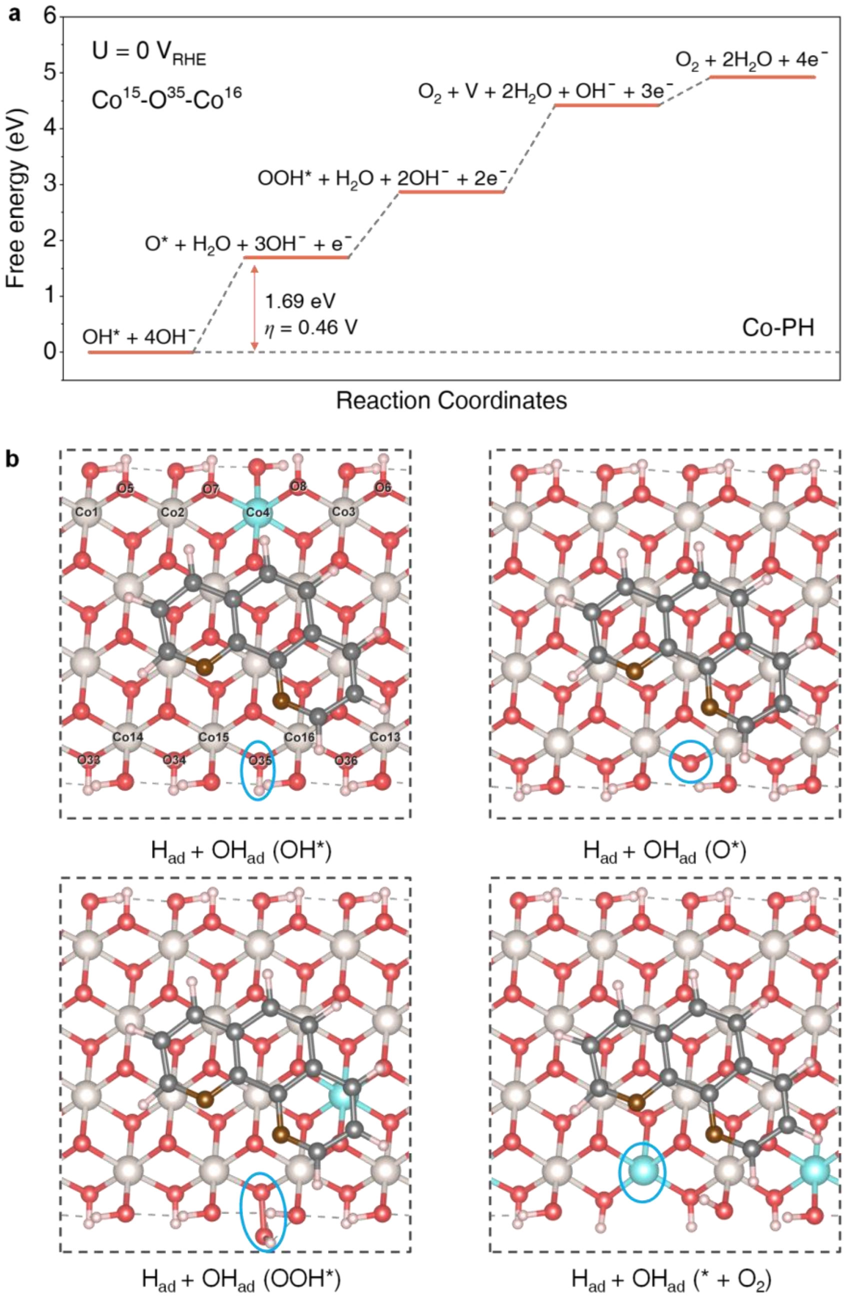

Supplementary Fig. 68 | Reaction free-energy diagram of the Co^{15}-O^{35}-Co^{16} reaction site on the Co-PH at 0 V_{RHE}. (a) The reaction free-energy diagram on the **Co^{15}-O^{35}-Co^{16}** reaction site on Co-PH and (b) the corresponding models. The reaction sites are shown with light blue circles. Co^{4+}, Co^{3+}, N, C, O, and H are represented by silver, cyan, chocolate, gray, red, and pink balls, respectively.

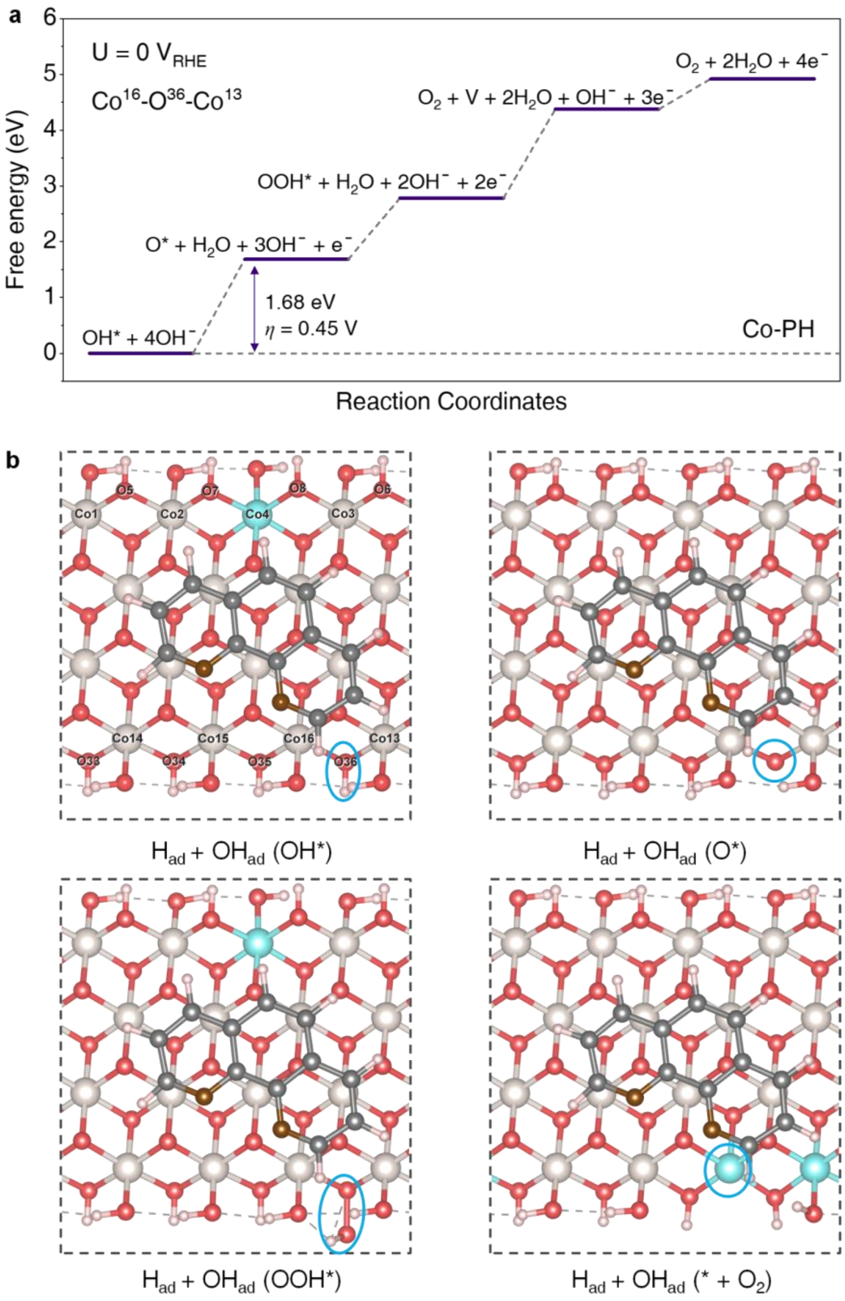

Supplementary Fig. 69 | Reaction free-energy diagram of the Co^{16}-O^{36}-Co^{13} reaction site on the Co-PH at 0 V_{RHE}. (a) The reaction free-energy diagram on the **Co^{16}-O^{36}-Co^{13}** reaction site on Co-PH and (b) the corresponding models. The reaction sites are shown with light blue circles. Co^{4+}, Co^{3+}, N, C, O, and H are represented by silver, cyan, chocolate, gray, red, and pink balls, respectively.

Supplementary References

1 Shimanouchi, T. *Tables of molecular vibrational frequencies, consolidated volume I*. NSRDS-NBS **39** (1972).

2 Huber, K. P. a. H., G. *Molecular spectra and molecular structure: IV Constants of diatomic molecules*. Van Nostrand Reinhold Co (1979).

3 Chase, M. *NIST-JANAF thermochemical tables*. 4th edn, American Institute of Physics (1998).

4 Zeng, Z. H. & Greeley, J. Characterization of oxygenated species at water/Pt(111) interfaces from DFT energetics and XPS simulations. *Nano Energy* **29**, 369-377 (2016).

5 Wang, L. *et al.* Core-shell nanostructured cobalt-platinum electrocatalysts with enhanced durability. *ACS Catal.* **8**, 35-42 (2018).

6 Dionigi, F. *et al.* In-situ structure and catalytic mechanism of NiFe and CoFe layered double hydroxides during oxygen evolution. *Nat. Commun.* **11**, 2522-2531 (2020).

7 Strmcnik, D. *et al.* The role of non-covalent interactions in electrocatalytic fuel-cell reactions on platinum. *Nat. Chem.* **1**, 466-472 (2009).

8 Muller-Dethlefs, K. & Hobza, P. Noncovalent interactions: a challenge for experiment and theory. *Chem. Rev.* **100**, 143-168 (2000).

9 Morisawa, Y., Ikehata, A., Higashi, N. & Ozaki, Y. Attenuated total reflectance-far ultraviolet (ATR-FUV) spectra of CH_3OH, CH_3OD, CD_3OH and CD_3OD in a liquid phase ~Rydberg states~. *Chem. Phys. Lett.* **476**, 205-208 (2009).

10 Higashi, N., Ikehata, A. & Ozaki, Y. An attenuated total reflectance far-UV spectrometer. *Rev. Sci. Instrum.* **78**, 103107-103112 (2007).

11 Kumar Trivedi, M. Thermal, spectroscopic and chromatographic characterization of Biofield energy treated benzophenone. *Sci. J. Anal. Chem.* **3**, 109-114 (2015)

12 Hoffmann, S. K., Goslar, J. & Lijewski, S. Electron paramagnetic resonance and electron spin echo studies of Co^{2+} coordination by nicotinamide adenine dinucleotide (NAD^+) in water solution. *Appl. Magn. Reson.* **44**, 817-826 (2013).

13 Kutin, Y., Cox, N., Lubitz, W., Schnegg, A. & Rudiger, O. In situ EPR characterization of a cobalt oxide water oxidation catalyst at neutral pH. *Catalysts* **9**, 926-936 (2019).

14 Dionigi, F. *et al.* Intrinsic electrocatalytic activity for oxygen evolution of crystalline 3d-transition metal layered double hydroxides. *Angew. Chem. Int. Ed.* **60**, 14446-14457 (2021).

15 Burke, M. S., Kast, M. G., Trotochaud, L., Smith, A. M. & Boettcher, S. W. Cobalt-iron (oxy)hydroxide oxygen evolution electrocatalysts: the role of structure and composition on activity, stability, and mechanism. *J. Am. Chem. Soc.* **137**, 3638-3648 (2015)

16 Yang, J., Liu, H. W., Martens, W. N. & Frost, R. L. Synthesis and characterization of cobalt hydroxide, cobalt oxyhydroxide, and cobalt oxide nanodiscs. *J. Phys. Chem. C* **114**, 111-119 (2010).

17 Petrie, J. R., Jeen, H., Barron, S. C., Meyer, T. L. & Lee, H. N. Enhancing perovskite electrocatalysis through strain tuning of the oxygen deficiency. *J. Am. Chem. Soc.* **138**, 7252-7255 (2016).

18 Dupin, J. C., Gonbeau, D., Benqlilou-Moudden, H., Vinatier, P. & Levasseur, A. XPS analysis of new lithium cobalt oxide thin-films before and after lithium deintercalation. *Thin Solid Films* **384**, 23-32 (2001).

19 Liu, B., Zhang, Y. & Tang, L. X-ray photoelectron spectroscopic studies of $Ba_{0.5}Sr_{0.5}Co_{0.8}Fe_{0.2}O_{3-\delta}$ cathode for solid oxide fuel cells. *Int. J. Hydrog. Energy* **34**, 435-439 (2009).

20 Meng, C. *et al.* Laser synthesis of oxygen vacancy-modified CoOOH for highly efficient oxygen evolution. *Chem. Commun.* **55**, 2904-2907 (2019).

21 Artyushkova, K. *et al.* Density functional theory calculations of XPS binding energy shift for nitrogen-containing graphene-like structures. *Chem. Commun.* **49**, 2539-2541 (2013).

22 Thornton, D. A. & Watkins, G. M. A full vibrational assignment (4000-50 cm^{-1}) of 1,10-phenanthroline and its perdeuterated analog. *Spectrochim. Acta A* **47**, 1085-1096 (1991).

23 Idir, B. & Kellou-Kerkouche, F. Experimental and theoretical studies on corrosion inhibition performance of phenanthroline for cast iron in acid solution. *J. Electrochem. Sci. Technol* **9**, 260-275 (2018).

24 Jagadale, A. D., Dubal, D. P. & Lokhande, C. D. Electrochemical behavior of potentiodynamically deposited cobalt oxyhydroxide (CoOOH) thin films for supercapacitor application. *Mater. Res. Bull.* **47**, 672-676 (2012).

25 Nagaraju, G., Ko, Y. H. & Yu, J. S. Self-assembled hierarchical beta-cobalt hydroxide nanostructures on conductive textiles by one-step electrochemical deposition. *Crystengcomm* **16**, 11027-11034 (2014).

26 Subbaraman, R. *et al.* Trends in activity for the water electrolyser reactions on 3d M(Ni,Co,Fe,Mn) hydr(oxy)oxide catalysts. *Nat. Mater.* **11**, 550-557 (2012).

27 Li, Y. *et al.* Bifunctional porous cobalt phosphide foam for high curren density alkaline water electrolysis with 4000 h long stability. *ACS Sustain. Chem. Eng.* **8**, 10193-10200 (2020).

28 Lee, C. *et al.* Atomically embedded Ag via electrodiffusion boosts oxygen evolution of CoOOH nanosheet arrays. *ACS Catal.* **10**, 562-569 (2020).

29 Zhang, S. L. *et al.* Metal atom-doped Co_3O_4 hierarchical nanoplates for electrocatalytic oxygen evolution. *Adv. Mater.* **32**, 2002235-2002242 (2020).

30 Liu, C. *et al.* Oxygen evolution reaction over catalytic single-site Co in a well-defined brookite TiO_2 nanorod surface. *Nat. Catal.* **4**, 36-45 (2021).

31 Song, F. & Hu, X. L. Exfoliation of layered double hydroxides for enhanced oxygen evolution catalysis. *Nat. Commun.* **5**, 4477-4485 (2014).

32 Song, F. & Hu, X. Ultrathin cobalt-manganese layered double hydroxide is an efficient oxygen evolution catalyst. *J. Am. Chem. Soc.* **136**, 16481-16484 (2014).

33 Dionigi, F. *et al.* In-situ structure and catalytic mechanism of NiFe and CoFe layered double hydroxides during oxygen evolution. *Nat. Commun.* **11**, 2522-2531 (2020).

34 Wang, Y. Y. *et al.* Layered double hydroxide nanosheets with multiple vacancies obtained by dry exfoliation as highly efficient oxygen evolution electrocatalysts. *Angew. Chem. Int. Ed.* **56**, 5867-5871 (2017).

35 Zhang, B. *et al.* Homogeneously dispersed multimetal oxygen-evolving catalysts. *Science* **352**, 333-337 (2016).

36 Zhang, B. *et al.* High-valence metals improve oxygen evolution reaction performance by modulating 3d metal oxidation cycle energetics. *Nat. Catal.* **12**, 2203-2210 (2020).

37 Guo, F. *et al.* High-performance oxygen evolution electrocatalysis by boronized metal sheets with self-functionalized surfaces. *Energy Environ. Sci.* **12**, 684-692 (2019).

38 Ping, J. F. *et al.* Self-assembly of single-layer CoAl-layered double hydroxide nanosheets on 3D graphene network used as highly efficient electrocatalyst for oxygen evolution reaction. *Adv. Mater.* **28**, 7640-7645 (2016).

39 Wang, J. *et al.* Redirecting dynamic surface restructuring of a layered transition metal oxide catalyst for superior water oxidation. *Nat. Catal.* **4**, 212-222 (2021).

40 Li, X. *et al.* Adaptive bifunctional electrocatalyst of amorphous CoFe oxide @ 2D black phosphorus for overall water splitting. *Angew. Chem. Int. Ed.* **59**, 2-10 (2020).

41 Dou, Y. *et al.* Approaching the activity limit of $CoSe_2$ for oxygen evolution via Fe doping and Co vacancy. *Nat. Commun.* **11**, 1664-1672 (2020).

42 He, P., Yu, X. Y. & Lou, X. W. Carbon-incorporated nickel-cobalt mixed metal phosphide nanoboxes with enhanced electrocatalytic activity for oxygen evolution. *Angew. Chem. Int. Ed.* **56**, 3897-3900 (2017).

43 Qian, Q. Z., Li, Y. P., Liu, Y., Yu, L. & Zhang, G. Q. Ambient fast synthesis and active sites deciphering of hierarchical foam-like trimetal-organic framework nanostructures as a platform for highly efficient oxygen evolution electrocatalysis. *Adv. Mater.* **31**, 1901139-1901146 (2019).

44 Yang, H. *et al.* Preparation of nickel-iron hydroxides by microorganism corrosion for efficient oxygen evolution. *Nat. Commun.* **11**, 5075-5083 (2020).
